# Parallel trends in cortical gray and white matter architecture and connections in primates allow fine study of pathways in humans and reveal network disruptions in autism

**DOI:** 10.1371/journal.pbio.2004559

**Published:** 2018-02-05

**Authors:** Basilis Zikopoulos, Miguel Ángel García-Cabezas, Helen Barbas

**Affiliations:** 1 Human Systems Neuroscience Laboratory, Department of Health Sciences, Boston University, Boston, Massachusetts, United States of America; 2 Graduate Program in Neuroscience, Boston University, Boston, Massachusetts, United States of America; 3 Neural Systems Laboratory, Department of Health Sciences, Boston University, Boston, Massachusetts, United States of America; Stanford University School of Medicine, United States of America

## Abstract

Noninvasive imaging and tractography methods have yielded information on broad communication networks but lack resolution to delineate intralaminar cortical and subcortical pathways in humans. An important unanswered question is whether we can use the wealth of precise information on pathways from monkeys to understand connections in humans. We addressed this question within a theoretical framework of systematic cortical variation and used identical high-resolution methods to compare the architecture of cortical gray matter and the white matter beneath, which gives rise to short- and long-distance pathways in humans and rhesus monkeys. We used the prefrontal cortex as a model system because of its key role in attention, emotions, and executive function, which are processes often affected in brain diseases. We found striking parallels and consistent trends in the gray and white matter architecture in humans and monkeys and between the architecture and actual connections mapped with neural tracers in rhesus monkeys and, by extension, in humans. Using the novel architectonic portrait as a base, we found significant changes in pathways between nearby prefrontal and distant areas in autism. Our findings reveal that a theoretical framework allows study of normal neural communication in humans at high resolution and specific disruptions in diverse psychiatric and neurodegenerative diseases.

## Author summary

Can the wealth of information from animal studies on the structure and connections of the cerebral cortex—the brain’s outer rim—be translated to understand neural communication in humans and disruption in brain diseases? To address this question, we examined the prefrontal cortex, which is associated with attention, emotions, and executive control—functions that are disrupted in psychiatric and neurologic diseases. We compared the architecture of the human and rhesus monkey cortex, using identical methods in both species to maximize the accuracy of comparisons. High-resolution microscopy revealed features of gray matter regions, made up of many cells, as well as of the white matter beneath, which contains axons that form connections between brain regions. We found that the architecture of the gray and white matter in humans and monkeys varies systematically (and in parallel) and reflects connections assessed by tracers. Using the template established with control human brains, we found significant differences in short- and long-distance pathways in the brains of individuals with autism. The framework established here helps predict patterns in the architecture and connections of areas across mammalian species and sets the stage to integrate functional imaging data from control subjects to compare with pathological states in humans.

## Introduction

The functional specialization of the human cerebral cortex is critically dependent on the structural organization and connectivity of its cortical areas [1–5]. In recent years, evidence to support this structure-function relationship has focused on noninvasive methods through functional imaging and tractographic studies in humans. These approaches have led to analysis of large datasets and findings on broad brain communication networks [6–8].

Notwithstanding the introduction of approaches with increasing resolution to probe structure-function relationships in recent years, little is known about the efficacy of image-based tracing methods to capture the wealth of existing connections in humans [9–12]. This drawback is particularly acute in depicting even strong pathways to and from small subcortical nuclei or distinct cortical layers (e.g., [13–19]) while avoiding emergence of false pathways [20].

By contrast, a wealth of high-resolution information on connections has been amassed in nonhuman primates using invasive neural tracing methods, which have contributed to theories about the organization of connections (reviewed in [21, 22]). Moreover, numerous studies of the cyto-, myelo-, and receptor-architecture have systematically quantified neocortical laminar patterns and correlated them with gene expression or activity patterns (e.g., [5, 23–26]; reviewed in [27]). One key principle that has emerged is that the architectonic differences in the cortex of mammalian species are not random but systematic. Moreover, connections critically depend on the systematic variation in cortical structure (reviewed in [21]).

Can we use the rich information from monkeys to understand connections in humans and then examine their disruption in disease? We addressed this issue using the prefrontal cortex (PFC) as a model system in monkeys and humans, because this region is affected disproportionally in psychiatric and neurological diseases. The overall organization of the PFC and associated white matter bundles appear to be largely preserved in primate evolution, rendering nonhuman primates an invaluable animal model for the study of connections in humans [1, 17, 19, 28–33].

Our goal was to compare first the fundamental architecture of distinct prefrontal areas in humans and monkeys and then their axons below the cortex, which make up the highway system for connections. The methods to address this issue in humans and monkeys were identical. We then used detailed connection data from monkeys to determine whether pathways studied through axon features at high resolution are correlated with actual connections, studied with neural tracers. We used the PFC as a model system because it has a large array of areas with distinct laminar structure and functions [34, 35], and there are extensive quantitative data on its connections in nonhuman primates (reviewed in [28, 36–40]).

Within the framework of systematic variation in the cortex, we studied 3 functionally and structurally distinct PFC regions: anterior cingulate cortex (ACC), posterior orbitofrontal cortex (OFC), and lateral prefrontal cortex (LPFC), which are broadly associated with attention, emotions, and executive function [41]. We provide evidence for a strong similarity in the architecture of the cortex and white matter axons that participate in short- and long-distance communication in monkeys and humans and a high correlation between axon features and actual connections in monkeys and, by extension, in humans. Against the template of the high correlation of axon features and connections, further analyses revealed a changed trajectory of axons in autism spectrum disorder (ASD), suggesting disruption in both nearby and distant neural communication. Lastly, guided by the rules linking connections with the systematic structural variation of the cortex, we provide a map of connections that can be used in future studies to test hypotheses regarding networks likely affected in ASD.

## Results

### Gray matter architecture revealed some differences but also common trends in monkeys and humans

**Fig 1:**
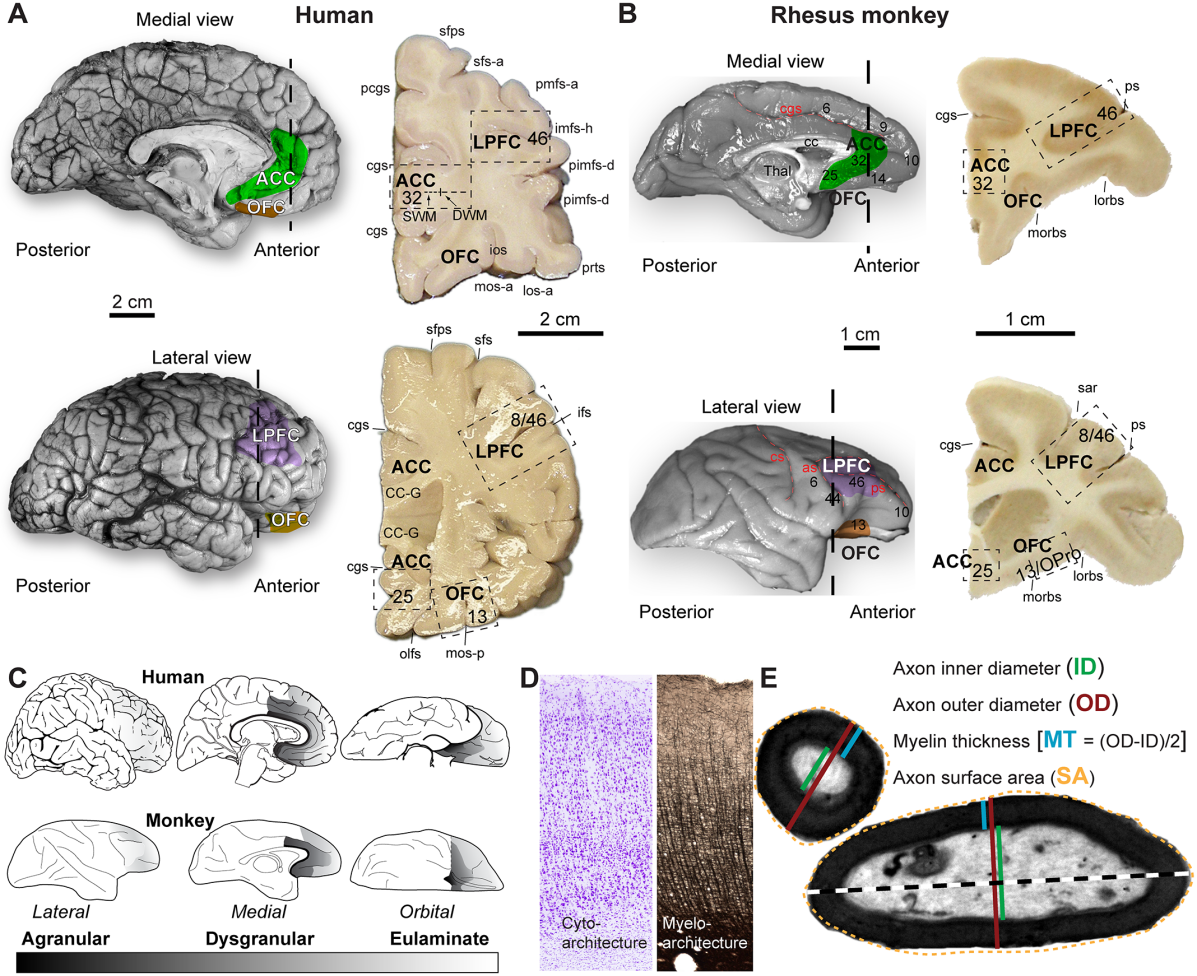
Experimental design and areas studied. (A) Human brain hemisphere (top-left, medial view; bottom-left, lateral view) and coronal tissue slabs (right) at 2 frontal levels (marked by dotted lines) with anterior cingulate cortex (ACC), orbitofrontal cortex (OFC), and lateral prefrontal cortex (LPFC) cortical regions, as well as superficial white matter (SWM) and deep white matter (DWM) subdivision beneath ACC. Dotted-line boxes outline the sampled columns. (B) Rhesus macaque brain hemisphere (top-left, medial view; bottom-left, lateral view) and coronal tissue slabs (right) at 2 frontal levels (marked by dotted lines) with corresponding ACC, OFC, and LPFC cortical regions. Dotted-line boxes outline the sampled columns. (C) Lateral, medial, and orbital views of hemispheres show prefrontal areas in the human (top) and rhesus macaque (bottom). The prefrontal cortex is shaded with a grayscale by structural type (agranular: dark shades, dysgranular: medium shades, and eulaminate: light shades). (D) Laminar cytoarchitecture and myeloarchitecture in cortical gray matter columns from coronal sections of ACC, OFC, and LPFC were studied quantitatively at the light microscope. (E) Estimation of axon and myelin thickness and density in the white matter beneath these prefrontal regions in the electron microscope. Color shading in panels A and B shows approximate regions of interest within ACC, OFC, and LPFC; the panels do not depict exact architectonic borders of these regions. We avoided regions near the borders of areas and sampled columns of dysgranular ACC, OFC, and granular LPFC (boxed outlines in coronal tissue slabs in panels A and B). Abbreviations: cc, corpus callosum; CC-G, corpus callosum genu; cgs, cingulate sulcus; ifs, inferior frontal sulcus; imfs-h, intermediate frontal sulcus, horizontal segment; ios, intermediate orbital sulcus; lorbs, lateral orbital sulcus; los-a, lateral orbital sulcus, anterior ramus; morbs, medial orbital sulcus; mos-a, medial orbital sulcus, anterior ramus; mos-p, medial orbital sulcus, posterior ramus; olfs, olfactory sulcus; pcgs, paracingulate sulcus; pimfs-d, paraintermediate frontal sulcus, dorsal; pmfs-a, posterior middle frontal sulcus, anterior segment; pmfs-i, posterior middle frontal sulcus, inferior segment; ps, principal sulcus; prts, pretriangular sulcus; sar, superior arcuate sulcus; sfps, superior frontal paramidline sulcus; sfs, superior frontal sulcus; sfs-a, superior frontal sulcus, anterior segment; Thal, thalamus.

**Fig 2:**
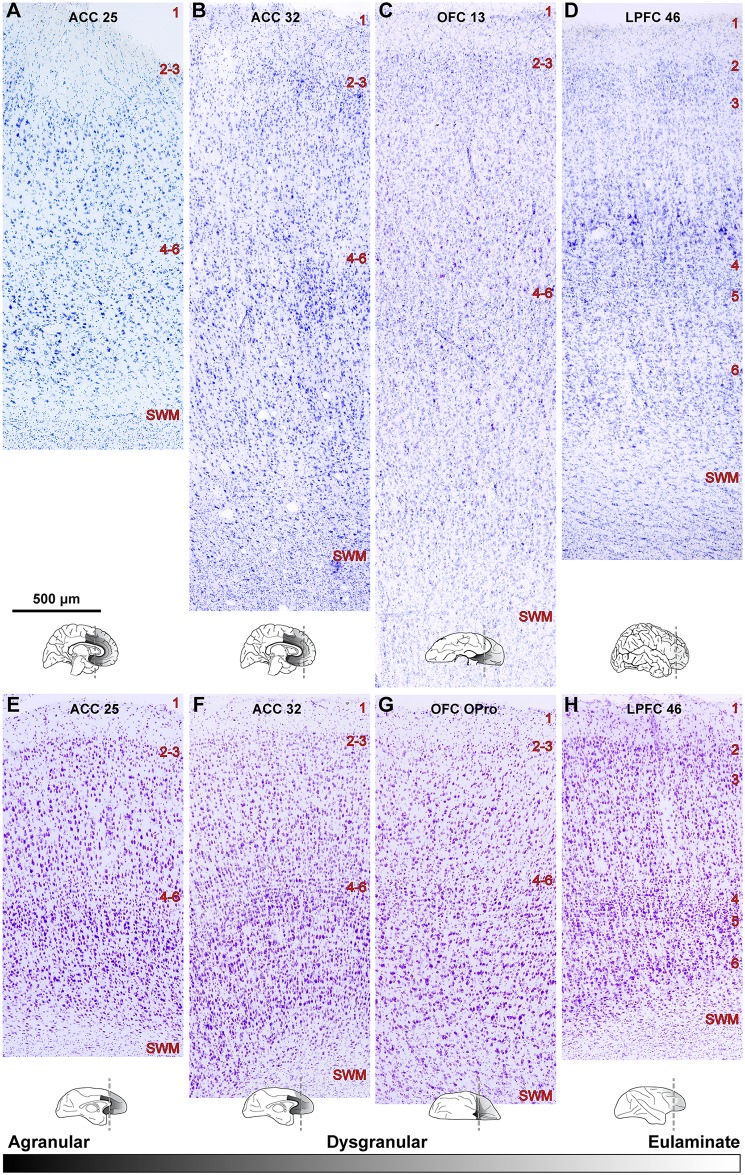
Distinct laminar cytoarchitecture of dysgranular (limbic) and eulaminate areas of the human and rhesus monkey prefrontal cortex (PFC). (A–D) Photomicrographs of coronal sections from areas 25, 32, and 13 (dysgranular) and area 46 (eulaminate) of the human brain stained with Nissl. (E–H) Photomicrographs of coronal sections from similar levels of areas 25, 32, orbital proisocortex (OPro) (level similar to human 13), and 46 of the rhesus macaque brain stained with Nissl. Small sketches under each panel show medial, lateral, and orbital views of the brain surface and the level of each coronal section. The sketches also show PFC areas with the lowest (black, agranular) and highest (lightest gray, eulaminate) laminar elaboration. Areas 25 and 32 have a rudimentary layer 4 (dysgranular). Deep layers 5–6 are more prominent than superficial layers 2–3 (A, B, E, and F). Eulaminate area 46 has 6 layers, with a well-developed layer 4. Superficial layers 2–3 are denser than in limbic anterior cingulate cortex (ACC) areas (D, H). Dysgranular area 13/OPro in the orbitofrontal cortex (OFC) shows intermediate characteristics (C, G). We examined OFC area 13 in human PFC and the structurally similar OFC area OPro in rhesus macaques. Abbreviations: LPFC, lateral prefrontal cortex; OPro, orbital proisocortex; SWM, superficial white matter. Red numbers indicate cortical layers. The calibration bar in panel A applies to all photographs.

Fig 1 shows the overall experimental design, and Tables 1–3 and S1 and S2 Tables include information on the humans and monkeys used in the present study. We first quantified and compared key cytoarchitectonic features of the PFC in both species, including the laminar density of neurons. The investigated ACC areas 25 and 32 and OFC areas 13 and orbital proisocortex (OPro) are dysgranular, an architectonic term applied to areas that have overall poor laminar definition and inconspicuous layer 4, as shown in Fig 2. We refer to these areas collectively as limbic, to describe operationally areas that are either dysgranular or agranular (lacking layer 4). LPFC areas 46 and 8 are eulaminate, a term used for areas that have 6layers, including a clearly visible layer 4 (Fig 2). These terms apply to all areas of the cerebral cortex, including primary motor area 4, which has been incorrectly described as “agranular” in the literature (for discussion, see [42, 43]).

**Table 1:** Clinical characteristics of human subjects analyzed.

Human subject number	Diagnosis	Sex (M: male; F: female)	Age (years)	PMI (hours)	Hemisphere (L: left; R: right)	Primary cause of death
HAW	Control	F	58	–	Right	Pancreatic cancer
HAY	Control	M	67	–	Right	Pancreatic cancer
HBJ	Control	M	50	–	Right	Unknown
HBK	Control	M	60	–	Right	Unknown
B-4786	Control	M	36	20	Right	Myocardial infarction
B-4981	Control	M	42	18	Right	Myocardial infarction
B-5353	Control	F	41	14	Right	Unknown
B-6004	Control	F	36	18	Right	Unknown
AN-06746^a^	Autism	M	44	31	Right	Acute myocardial infarction
AN-18892^b^	Autism	M	31	99	Right	Shooting
AN-08792^c^	Autism	M	30	20	Right	Gastrointestinal bleeding
AN-07770	Autism	F	40	33	Left	Respiratory arrest
AN-11989	Autism	M	30	16	Right	Congestive heart failure

Abbreviations: PMI, postmortem interval. Other diagnosed disorders include the following:

^a^ schizophrenia;

^b^ depression;

^c^ seizures.

**Table 2:** Adult rhesus monkeys analyzed.

Rhesus monkeys	Sex (M: male; F: female)	Age (years)	Hemisphere (L: left; R: right)
MBH	–	–	R
MDL	–	–	R
AA	F	–	R
AD	–	–	R
AF	–	–	L
AI	M	2	L + R
AJ	F	–	R
AK	F	3	R
AL	–	–	R
AN	–	–	L + R
AR	–	3	L + R
AS	F	5	L + R
AT	F	2	L + R
AV	–	–	L + R
AY	F	3	L
BB	F	2	L + R
BD	M	2.5	L + R
BF	F	2	R
BI	F	3	R
BJ	F	2	R
BL	M	3	R
BN	M	2	L + R
BS	F	3.5	L + R
BT	F	4	L + R
BU	M	4	L + R

**Table 3:** Type of experiment and analyses performed for each of the human subjects and each of the rhesus monkeys.

Human subjects	EM	Nissl	Myelin	Tract tracing
HAW	*	*	*	
HAY	*	*	*	
HBJ		#	#	
HBK		#	#	
B-4786	*	*		
B-4981	*	*		
B-5353	*	*		
B-6004	*	*		
AN-06746	*			
AN-18892	*			
AN-08792	*			
AN-07770	*			
AN-11989	*			
**Rhesus monkeys**				
MBH				*
MDL				*
AA				*
AD				*
AF				*
AI		#	#	
AJ			#	*
AK			#	*
AL			#	*
AN		#	#	
AR		*	*	
AS		*	*	
AT		*	*	
AV		*	*	
AY		*	*	*
BB		#		
BD		#		
BF				*
BI	*			*
BJ		#		
BL	*			
BN	*	#		
BS		#		
BT		#		
BU	*			

Abbreviations: EM, electron microscope. Asterisks (*) indicate human subjects and rhesus monkeys used for quantitative analyses using unbiased sampling or stereology. Pound signs/hashtags (#) indicate human subjects and rhesus monkeys used for qualitative or semiquantitative microscopic analyses.

We delineated areas based on the detailed descriptions and maps by von Economo, Koskinas, and Sanides [35, 44, 45] for the human cerebral cortex and Barbas and Pandya [38] for the rhesus macaque PFC. For this study, we avoided regions near the borders of areas, as described in recent detailed studies on the architecture of PFC regions [46–49], and relied on multiple salient features used in classic and modern architectonic studies to determine what constitutes an architectonic area, despite focal architectonic variations seen particularly across large areas. A good example of this approach is the characterization and mapping of area 25 in the rhesus macaque, which was based on cyto- and myeloarchitecture, as well as the distribution of neurofilament markers (SMI-32) and calcium-binding proteins [50]. The position of area 25 in the posterior, ventromedial (subgenual gyrus), and orbital surface of the PFC is comparable in rhesus monkeys and humans ([51], mainly areas F_L_ and F_H_ in the human cortical atlas by von Economo and Koskinas [44]). There is a gradual increase in the laminar elaboration of area 25 along the posterior to anterior and medial to lateral axes such that its most posterior and medial portions are agranular, whereas the anterior and lateral segments are dysgranular [35, 44, 50–52]. The salient and most distinguishing feature of area 25 across its entire extent is the increased thickness and neuronal density of deep layers 5 and 6 and the very low density of myelinated axons in both primate species [35, 50]. Here we focused on the dysgranular portion of area 25 for analysis. Gradual changes in the laminar architecture of the cortex have also been described in the OFC, where layer 4 is sparser in area OPro than in the rostrally situated area 13, which is also dysgranular [44, 53–56], and in the LPFC, where eulaminate area 8 is progressively more granular than the anteriorly situated area 46, which is also eulaminate [29, 30, 38, 44, 57–59].

**Fig 3:**
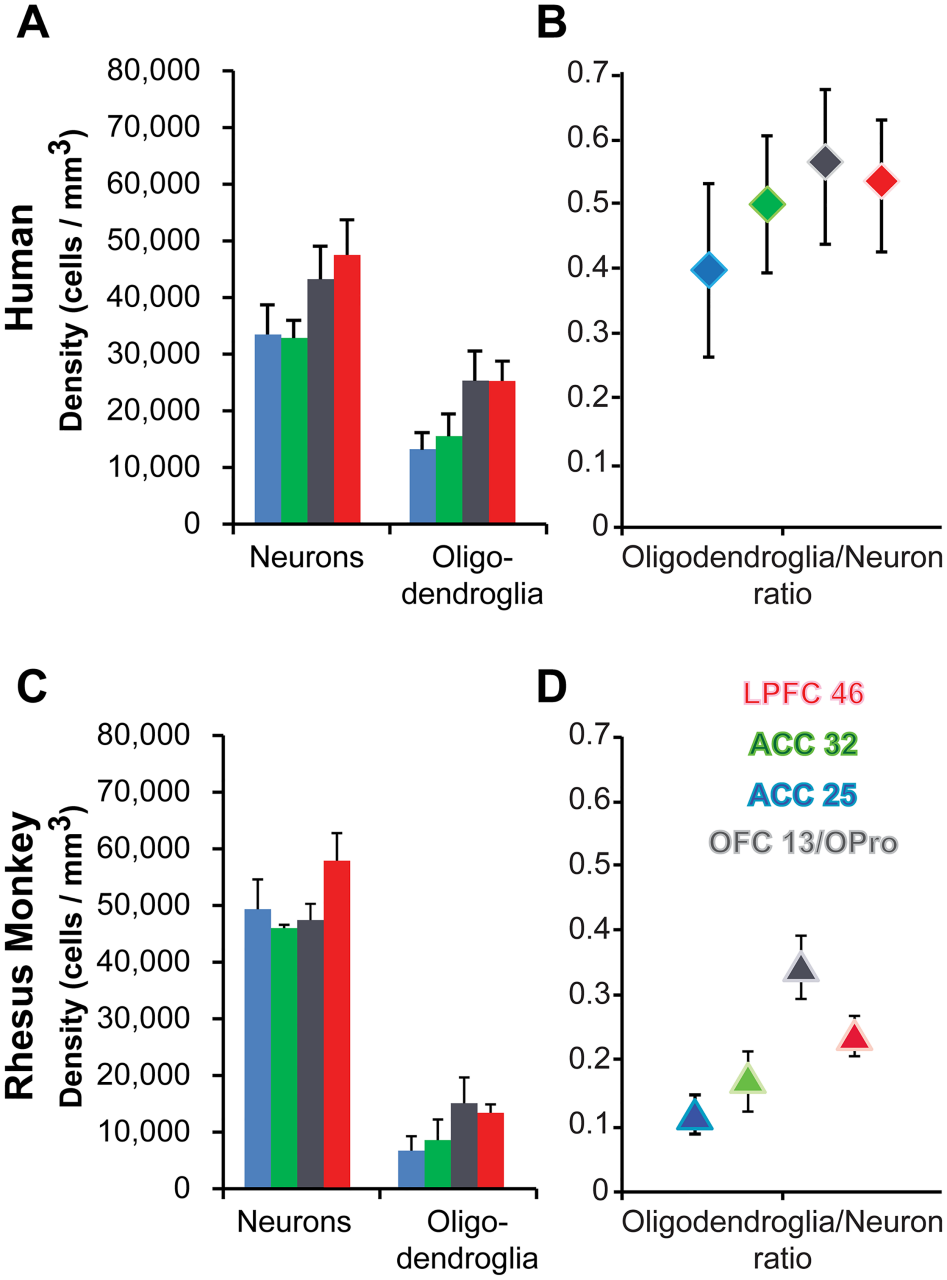
Quantitative cytoarchitecture of the human and rhesus monkey prefrontal cortex (PFC). (A, B) Human PFC. (C, D) Rhesus monkey PFC. (A) The density of neurons and oligodendroglia and (B) the oligodendroglia/neuron ratio in human PFC increase gradually from limbic anterior cingulate cortex (ACC) to orbitofrontal cortex (OFC) to eulaminate lateral prefrontal cortex (LPFC). (C) The density of neurons and oligodendroglia and (D) the oligodendroglia/neuron ratio in monkey PFC also show a similar, albeit less pronounced, gradual increase from limbic ACC to OFC to eulaminate LPFC. All values are mean ± standard error. Red, LPFC area 46; gray, OFC area 13/orbital proisocortex (OPro); green, ACC area 32; blue, ACC area 25. We examined OFC area 13 in human PFC and the structurally similar OFC area OPro in rhesus macaques. The numerical data underlying this figure can be found in S1 Data.

**Fig 4:**
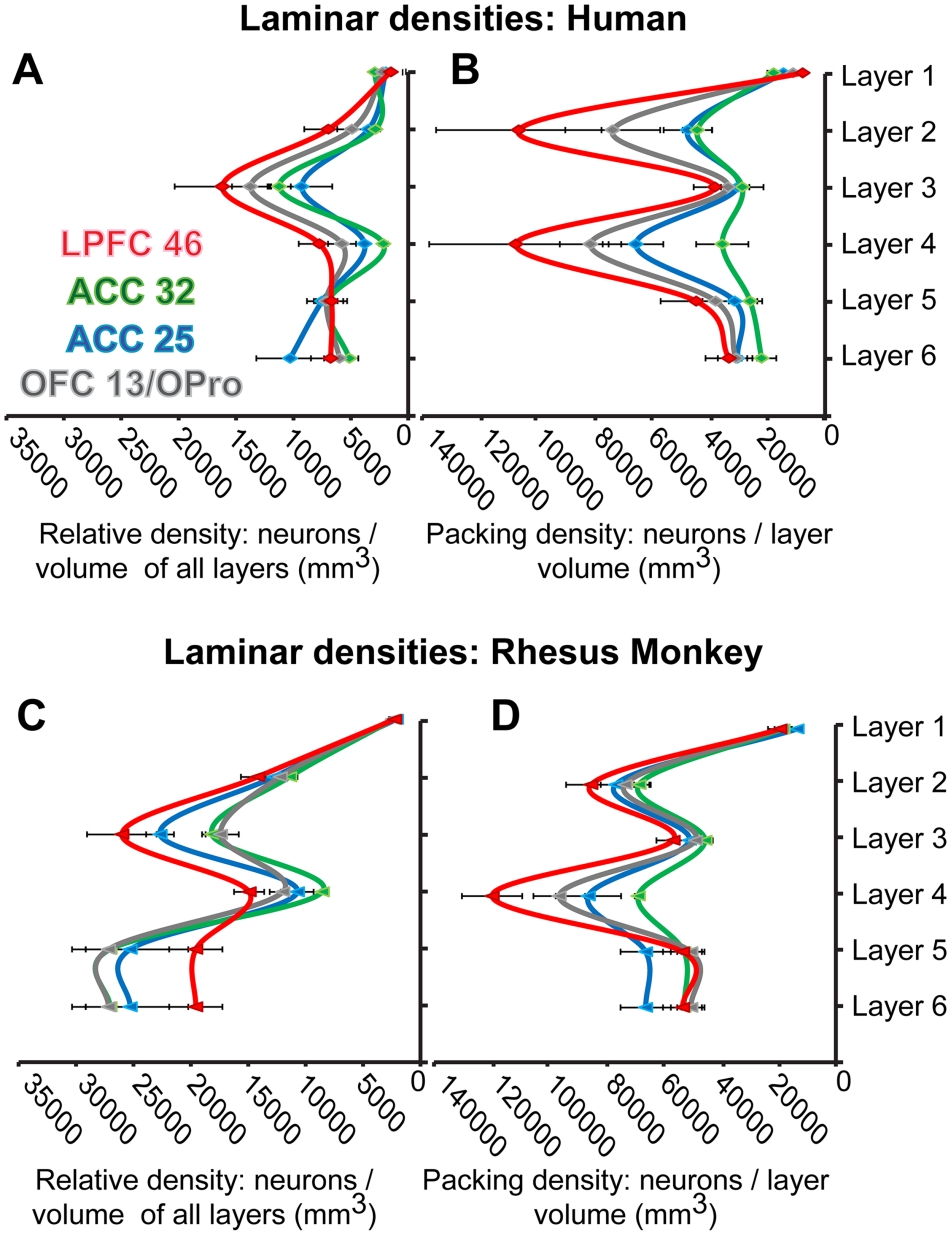
Quantitative laminar cytoarchitecture. (A, B) Human prefrontal cortex (PFC). (C, D) Rhesus monkey PFC. The relative laminar density of neurons in panels A and C was estimated as the average density of neurons in each layer divided by the volume (mm^3^) of all layers within a column of cortical gray matter. (A, C) show that most neurons are found in the superficial layers, especially layer 3, of eulaminate lateral prefrontal cortex (LPFC). In the anterior cingulate cortex (ACC), more neurons are found in deep layers 5 and 6 than in the upper layers, while the orbitofrontal cortex (OFC) shows intermediate features. In panels B and D, the packing laminar density of neurons was estimated as the average density of neurons in each layer divided by the volume (mm^3^) of that layer. This type of estimated density shows which layers have more densely packed populations of neurons within a cortical gray matter column of set volume. Neurons are densely packed in layer 2 and are especially so in layer 4. This is particularly evident in LPFC; the peaks of densely packed neurons become less prominent in other PFC areas, especially the ACC. All values are mean ± standard error. Red, LPFC area 46; gray, OFC area 13/orbital proisocortex (OPro); green, ACC area 32; blue, ACC area 25. The numerical data underlying this figure can be found in S1 Data.

Quantitative comparison of cytoarchitectural features showed that despite differences between humans and monkeys, the differences between limbic and eulaminate PFC areas followed similar trends in the 2 species. Human PFC areas had overall lower neuron density compared to rhesus macaques (Figs 2 and 3), but the relationship among areas was the same (Fig 3). Most neurons within a unit volume of human PFC (relative density) were found in layer 3, and neurons were most densely packed in layers 2 and 4, whereas in rhesus macaques neuron distribution and relative density were more balanced among layers, with the highest packing density seen in layer 4, as in the human (Fig 4). Pyramidal projection neurons in layers 3 and 5 were overall larger in humans.

Limbic and eulaminate PFC cortices with their distinct cytoarchitecture had differences in the density of neurons that followed similar trends across prefrontal areas in monkeys and humans (Figs 3 and 4). As shown in Fig 4, ACC cortices, and especially area 25, had relatively more neurons in deep layers 5 and 6, whereas LPFC had more neurons in the superficial layers. Fig 4 also shows that the packing density (neurons/layer volume in mm^3^) followed the same trend across areas in monkeys and humans. LPFC areas stand out by having 2 peaks in the density of neurons centered on layers 2 and 4, consistent with qualitative classic studies and earlier quantitative studies [34, 38, 45]. The OFC had an intermediate density of neurons, about equally distributed in the infragranular and supragranular layers.

The relative size of pyramidal projection neurons in layers 3 and 5 was also different among PFC areas. A characteristic feature of ACC was the presence of relatively large pyramidal neurons in layer 5, whereas in LPFC the largest neurons were found at the bottom of layer 3 (Fig 2). Once again, OFC had a more balanced appearance with slightly larger neurons in infragranular layer 5.

We then studied myelin in the gray matter, which coats a large number of axons in the primate cortex. First used in classic studies, myelin is an extremely useful feature for delineating architectonic areas, including the often misunderstood primary motor cortex (M1) in primates, which is heavily myelinated, as are other eulaminate areas [43]. In addition, myelin may be used to detect differences across cortical regions in the living human brain by imaging [60]. We also studied the density of oligodendrocytes, which myelinate axons.

**Fig 5:**
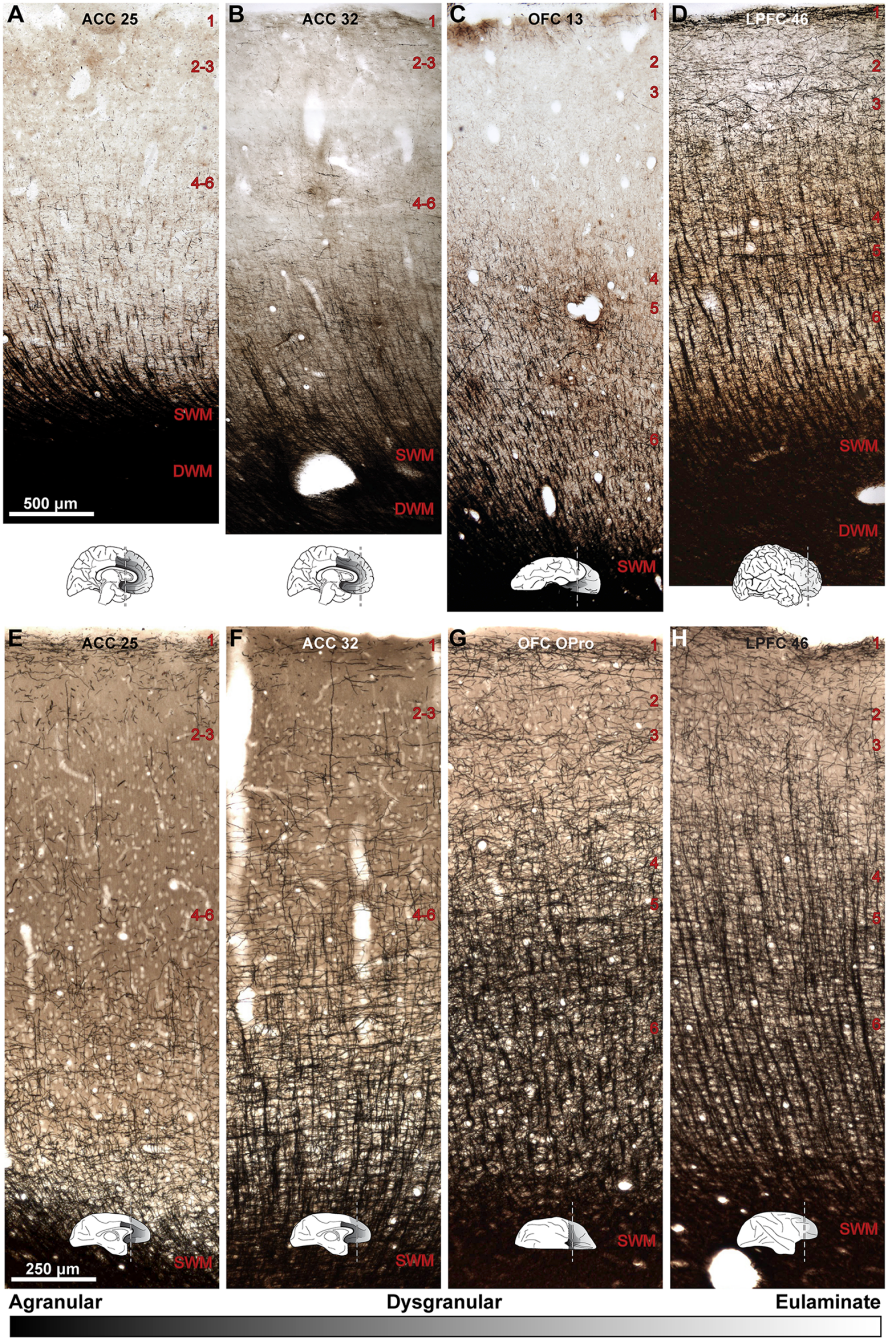
Distinct laminar myeloarchitecture of limbic and eulaminate areas of the human and rhesus monkey prefrontal cortex (PFC). (A–D) Photomicrographs of coronal sections from areas 25, 32, 13, and 46 of the human brain stained with the Gallyas technique for myelin. (E–H) Photomicrographs of coronal sections from similar levels of areas 25, 32, orbital proisocortex (OPro), and 46 of the rhesus macaque brain stained with the Gallyas technique for myelin. Small sketches under each panel show medial, lateral, and orbital views of the brain surface and the level of each coronal section. The sketches also show PFC areas with the lowest (black, agranular) and highest (lightest gray, eulaminate) laminar elaboration. There is a progressive increase of intracortical myelin from anterior cingulate cortex (ACC) to orbitofrontal cortex (OFC) to lateral prefrontal cortex (LPFC). ACC area 32 had slightly higher myelin levels compared to area 25, with a plexus of horizontal and vertical arrays of myelinated axons prominent in layers 4–6. The OFC had intermediate-to-high levels of myelin content, with a dense plexus of horizontal, vertical, and diagonal arrays of myelinated axons reaching layer 3. LPFC had the highest density of myelinated axons, with well-defined, dense columnar arrays of myelinated axons that reached layer 2. Human OFC area 13 is structurally similar to OFC area orbital proisocortex (OPro) in rhesus macaques. Abbreviations: DWM, deep white matter; SWM, superficial white matter. Red numbers indicate cortical layers. The calibration bar in panel A applies to panels A–D. The calibration bar in panel E applies to panels E–H.

**Fig 6:**
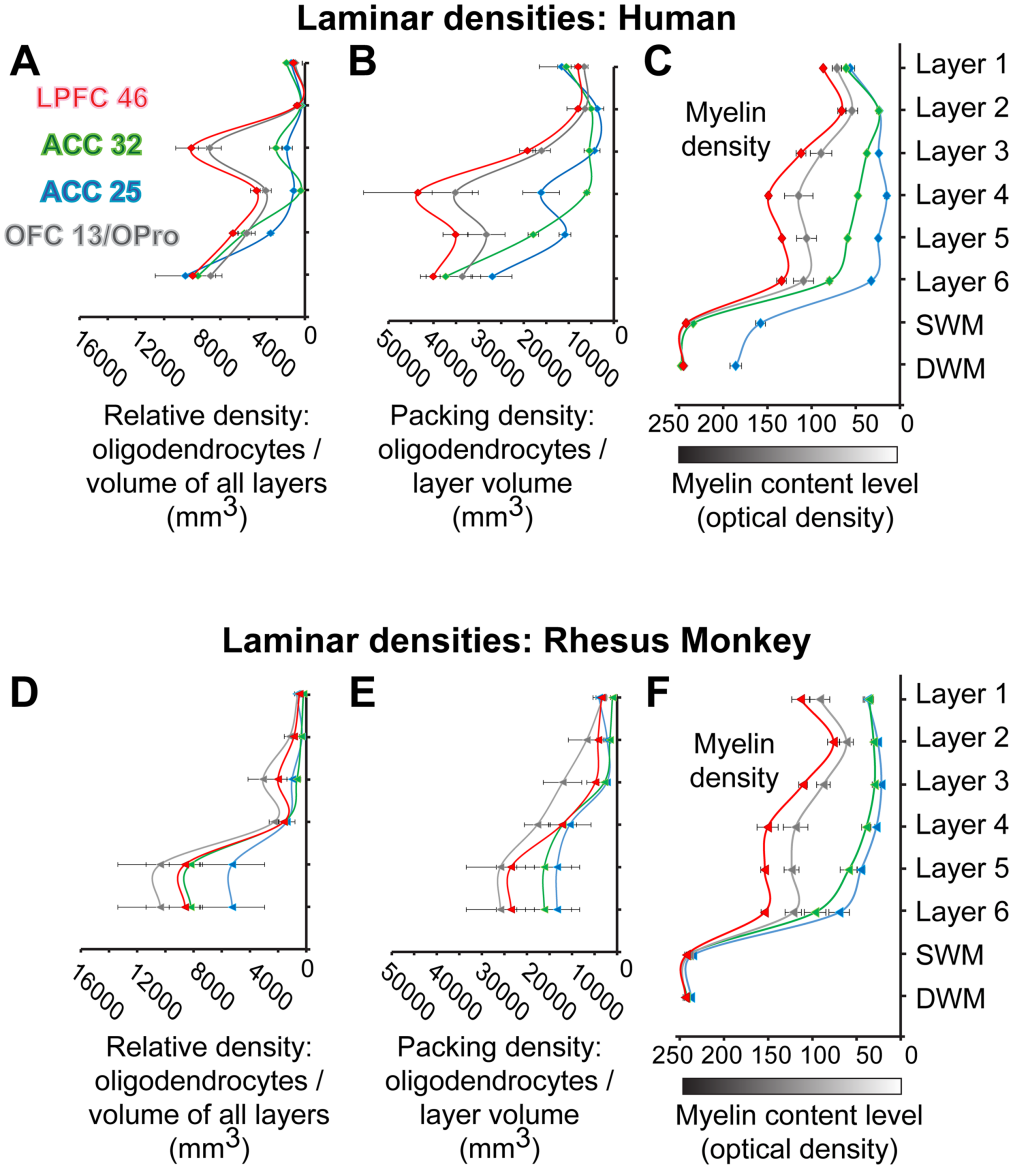
The laminar density of oligodendrocytes parallels the myelin content in limbic and eulaminate areas. (A–C) Human prefrontal cortex (PFC). (D–F) Rhesus monkey PFC. The relative laminar density of oligodendrocytes in panels A and D was estimated as the average density of oligodendrocytes in each layer divided by the volume (mm^3^) of all layers within a cortical gray matter column. Most oligodendrocytes are found in deep layers 5 and 6. The packing laminar density of oligodendrocytes in panels B and E was estimated as the average density of oligodendrocytes in each layer divided by the volume (mm^3^) of that layer. Oligodendrocytes are densely packed in layers 4–6 in all areas. The mean gray level index of myelin through the depth of the cortex also shows this trend in humans (C) and monkeys (F). Myelin content increased towards the white matter in the 4 areas. All values are mean ± standard error. Red, lateral prefrontal cortex (LPFC) area 46; gray, orbitofrontal cortex (OFC) area 13/orbital proisocortex (OPro); green, anterior cingulate cortex (ACC) area 32; blue, ACC area 25. Abbreviations: DWM, deep white matter; SWM, superficial white matter. The numerical data underlying this figure can be found in S1 Data.

The least myelinated areas were within the ACC, and the most myelinated areas were within LPFC, with OFC areas showing a pattern between the 2 extremes (Figs 5 and 6). Quantitative analysis showed that myelin density was highly and positively correlated with the laminar density of oligodendroglia in both species (Fig 6; R^2^ = 0.56), consistent with the role of oligodendroglia in myelinating axons. There were no significant differences in the myeloarchitecture of PFC in monkeys and humans, so the following description applies to both. Optical density measurements of the gray matter level index showed that myelin was relatively high in layer 1 and the transition from layer 5 to layer 6 and to the white matter in all areas examined (Fig 6). ACC area 25 had overall the lowest levels of myelin staining among the areas examined. Human PFC areas had overall higher oligodendrocyte density compared to rhesus macaques (Figs 3 and 6).

### White matter architecture revealed common trends in monkeys and humans

The above analyses revealed that humans and rhesus monkeys show common trends in the cytoarchitecture and myeloarchitecture of the gray matter across prefrontal areas. We then addressed whether this pattern extends to the architecture of axons beneath the areas studied in the 2 species. The significance of this analysis is based on the fact that axons make up pathways that connect cortical areas. We thus systematically examined the density and thickness of individual myelinated axons in ultrathin sections of the white matter beneath ACC, OFC, and LPFC at very high resolution with an electron microscope (EM). Myelinated axons made up about 50% of the white matter beneath the areas studied (range: 35%–60%). The remaining space was taken up primarily by unmyelinated axons and glia, especially oligodendrocytes.

**Fig 7:**
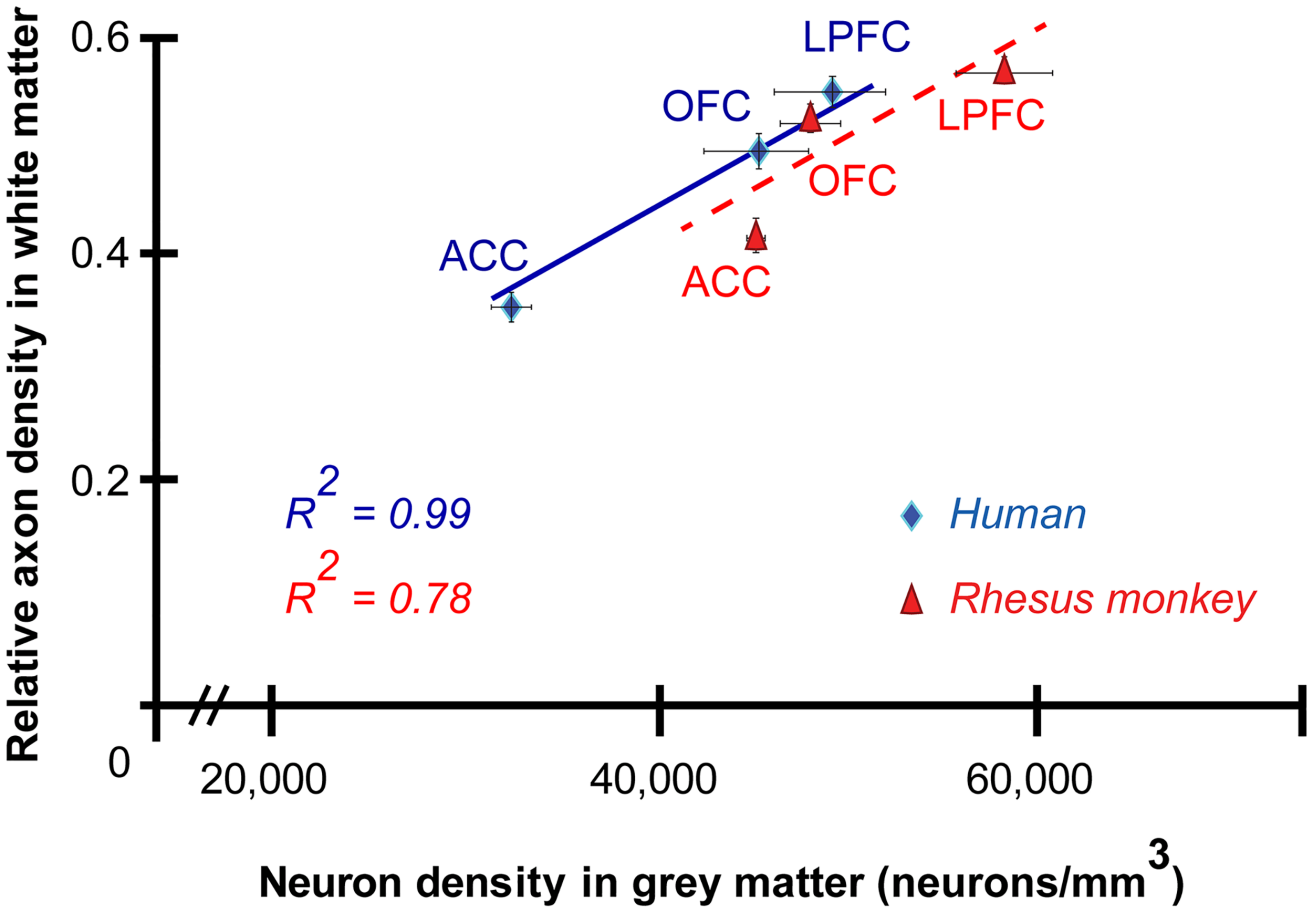
Consistent relationship of gray and white matter architecture. Neuron density in the prefrontal cortex (PFC) gray matter is increased in parallel to the density of myelinated axons in the white matter (including superficial white matter [SWM] and deep white matter [DWM]). The density of myelinated axons in the white matter and neurons in the gray matter gradually increased from anterior cingulate cortex (ACC) to orbitofrontal cortex (OFC) to lateral prefrontal cortex (LPFC) in both species (positive correlation R^2^ = 0.78, monkey; R^2^ = 0.99, human). Average values ± standard error are shown. Blue line and diamonds, human; red dotted line and triangles, rhesus monkey. The numerical data underlying this figure can be found in S1 Data.

As shown in Fig 7, there was a striking positive correlation between the density of neurons in the overlying gray matter and the density of myelinated axons in the white matter. Significantly, in both humans and monkeys, we found a gradually increasing trend from ACC to OFC to LPFC in the density of myelinated axons in the white matter and neurons in the gray matter (R^2^ = 0.8, monkey; R^2^ = 0.96, human).

**Fig 8:**
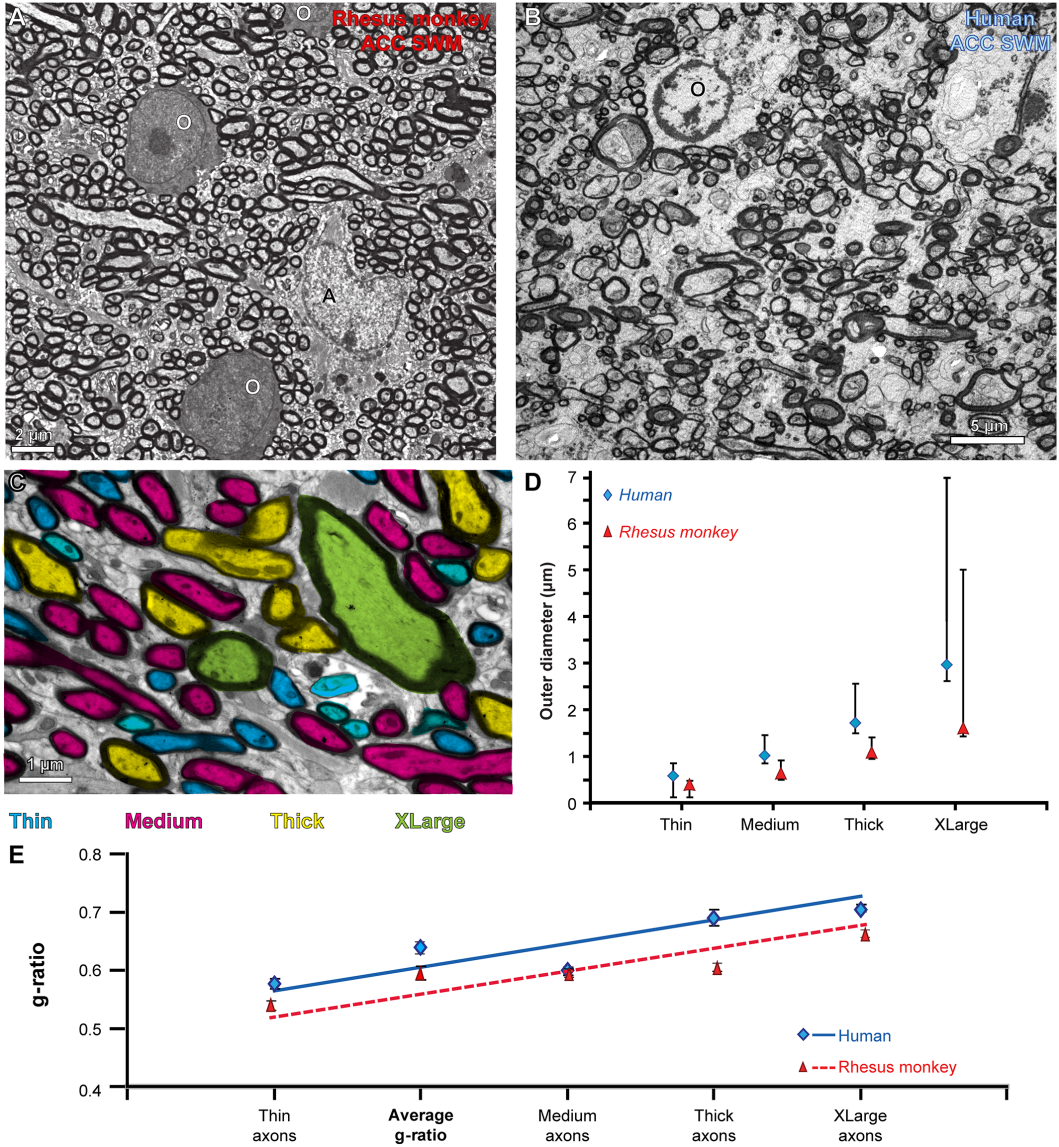
Architecture of myelinated axons in the white matter of the rhesus monkey and human prefrontal cortex (PFC). (A) Electron microscope (EM) photomicrograph of axon profiles in the superficial white matter below the anterior cingulate cortex (ACC) of a rhesus macaque. Two oligodendrocytes (O) and an astrocyte (A) are also visible. (B) EM photomicrograph of axon profiles in the superficial white matter below the ACC of an adult human. An oligodendrocyte (O) is also visible. (C) High magnification of an EM photomicrograph with circular and elongated profiles of axons color-coded according to their thickness (cyan, thin axons; magenta, medium axons; yellow, thick axons; green, extra-large axons). (D) Cluster analysis segregated axons into 4 groups based on their outer diameter (including myelin). Human axons were on average thicker than axons in rhesus monkeys. The graph shows size ranges (mean ± minimum and maximum diameter) for each axon size group. (E) G-ratio plot (inner/outer axon diameter ± standard error) in all areas and species examined. The average g-ratio of all axons in both species was near the optimal value (approximately 0.6). Thicker axons had larger g-ratio values compared to thinner axons. In panels D and E: blue line and diamonds, human; red dotted line and triangles, rhesus monkey. Abbreviations: SWM, superficial white matter. The numerical data underlying this figure can be found in S1 Data.

We next probed additional axon features in the white matter, in view of the fact that axons vary in thickness, a feature that affects axon dynamics, including the well-known differences in conduction velocity of neural impulses [61, 62]. Axon diameters below prefrontal areas ranged between 0.1–7 μm, in line with previous studies in human and macaque cortex [63–66]. The thickness of axons overlapped extensively but extended further within the large end of the spectrum in the human PFC (0.1–7 μm) than in rhesus macaques (0.1–5 μm; Fig 8A–8D). The finding of thicker axons in humans is consistent with the greater distances that axons must travel in the larger human brain, as well as the size of neurons in the 2 species.

As shown in Fig 8E, there was a positive linear correlation between the thickness of axons and the thickness of their myelin sheath. This finding is consistent with the classic relationship of the inner to outer diameter of axons, known as the g-ratio, which is an indicator of the efficiency of conduction velocity and neurotransmission. In all areas, the g-ratio increased significantly with axon size. In both species, the average g-ratio of axons was near the optimal average value (approximately 0.6).

We then used cluster analysis to segregate axons into 4 groups by thickness (Fig 8C and 8D), based on their outer diameters, which included the myelin sheath. Cluster analysis was conducted separately for monkeys and humans because of the differences in the thickness of axons between the 2 species. The cluster analysis separated axons in macaque monkeys into thin (0.1–0.49 μm), medium (0.5–0.86 μ m), thick (0.87–1.4 μm), and extra-large (larger than 1.4 μm) axons. In humans, cluster analysis similarly grouped axons into thin (0.1–0.83 μm), medium (0.84–1.51 μm), thick (1.52–2.65 μm), and extra-large (larger than 2.65 μm).

### Differentiating short- and long-range prefrontal pathways by axon thickness

**Fig 9:**
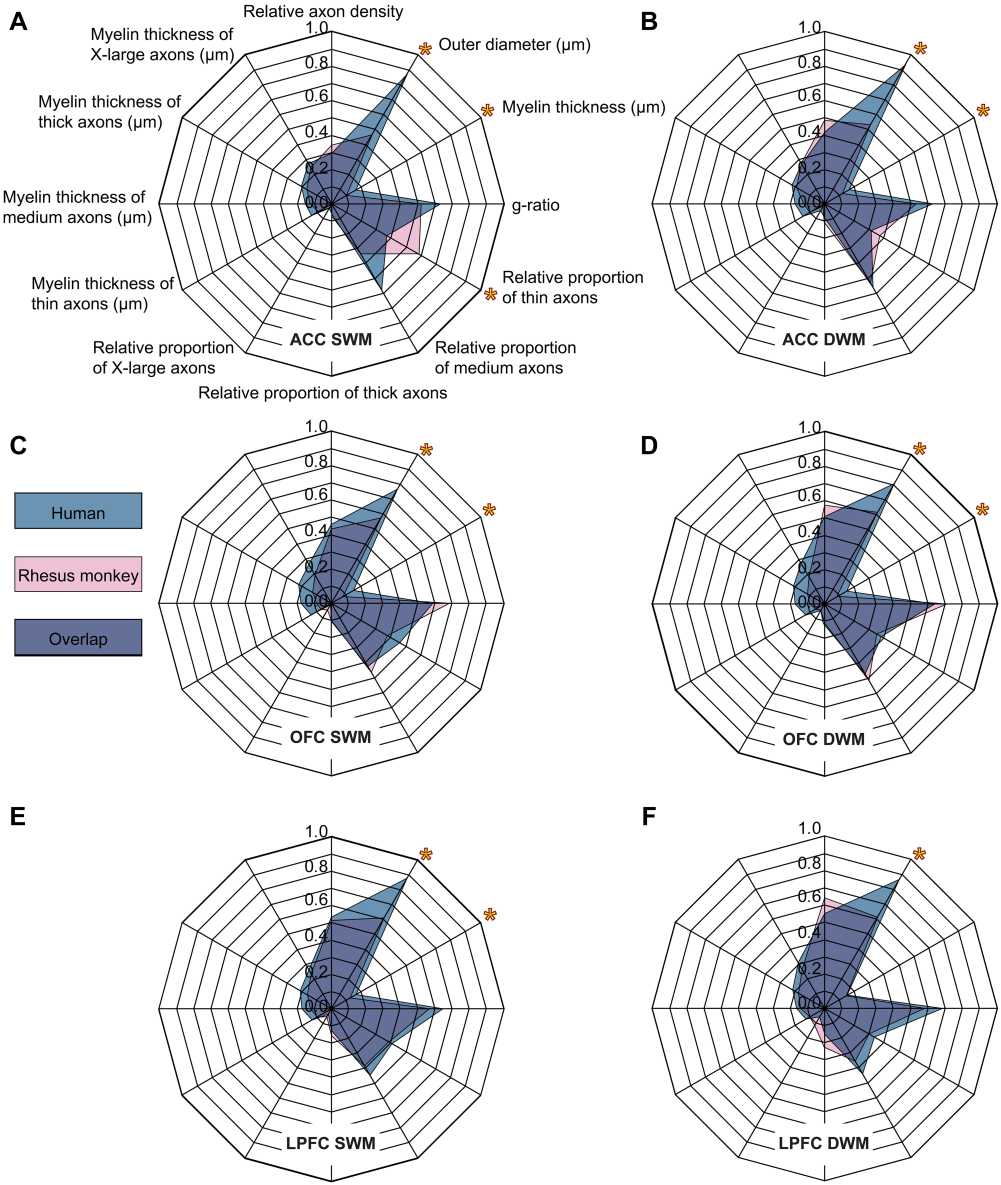
Fingerprints of areas based on structural features of axons in the white matter and their relative density identify distinct prefrontal areas in the rhesus monkey and human prefrontal cortex (PFC). (A–F) Fingerprint plots of the superficial white matter (SWM) and deep white matter (DWM) below anterior cingulate cortex (ACC) area 32, orbitofrontal cortex (OFC) area 13/ orbital proisocortex (OPro), and lateral prefrontal cortex (LPFC) area 46. *Significant differences between species (*p* < 0.05). The numerical data underlying this figure can be found in S1 Data.

In both humans and monkeys, thin and medium axons were the most numerous. Thick or extra-large axons constituted fewer than 30% of all axons beneath all PFC areas (Fig 9).

The relative position of axons within the white matter and overall diameter are also indicators of their termination in nearby or distant brain areas. Thus, thin axons in the outer white matter, near cortical layer 6, link nearby areas. This is consistent with 2 structural principles. First, as axons approximate their destination to innervate the cortex, they split into thinner terminal axons, akin to the thickness of the human forearm in comparison with the fingers. Second, fibers that link nearby areas follow the shortest route to their cortical destination within the superficial white matter, consistent with the principle of economy of wiring [67, 68]. By extension, thicker axons dive through the deep white matter as they travel to farther destinations [61, 69].

We used all structural parameters of axons to create unique fingerprints of the white matter in humans and monkeys for the 3 prefrontal regions, and the results are seen in Fig 9. The superficial white matter (SWM), which extended about 2 mm below layer 6, had relatively more thin axons compared to the deep white matter (DWM), which had relatively thicker axons in all areas and in both species.

As seen in the fingerprint diagrams, the similarities between the 2 species outnumber the differences, which were restricted to the outer diameter of axons in all areas, as well as myelin thickness across all areas except for LPFC (Fig 9; asterisks show statistically significant differences between humans and monkeys). Another species difference was in the SWM below ACC, which had a higher proportion of thin axons in rhesus monkeys than in humans (Fig 9A, lower asterisk).

In both species, the ACC and, to a lesser extent, the OFC had relatively more medium and thin axons compared to LPFC, especially in the superficial parts of the white matter, which contain mainly short- and medium-range pathways (Fig 9). In contrast, below LPFC there were relatively more thick to extra-large axons, especially in the DWM, which contains long-distance pathways (Fig 9). It should be noted that the SWM contains some thick axons as well, since pathways destined to travel over long distances must pass through the SWM in order to enter the DWM. The SWM also contains U-shaped fibers that connect neighboring gyri [28, 70], though these appear to be sparser than previously thought [71].

### Gray and white matter architecture is a sensitive indicator of interspecies and interareal similarities and differences

**Fig 10:**
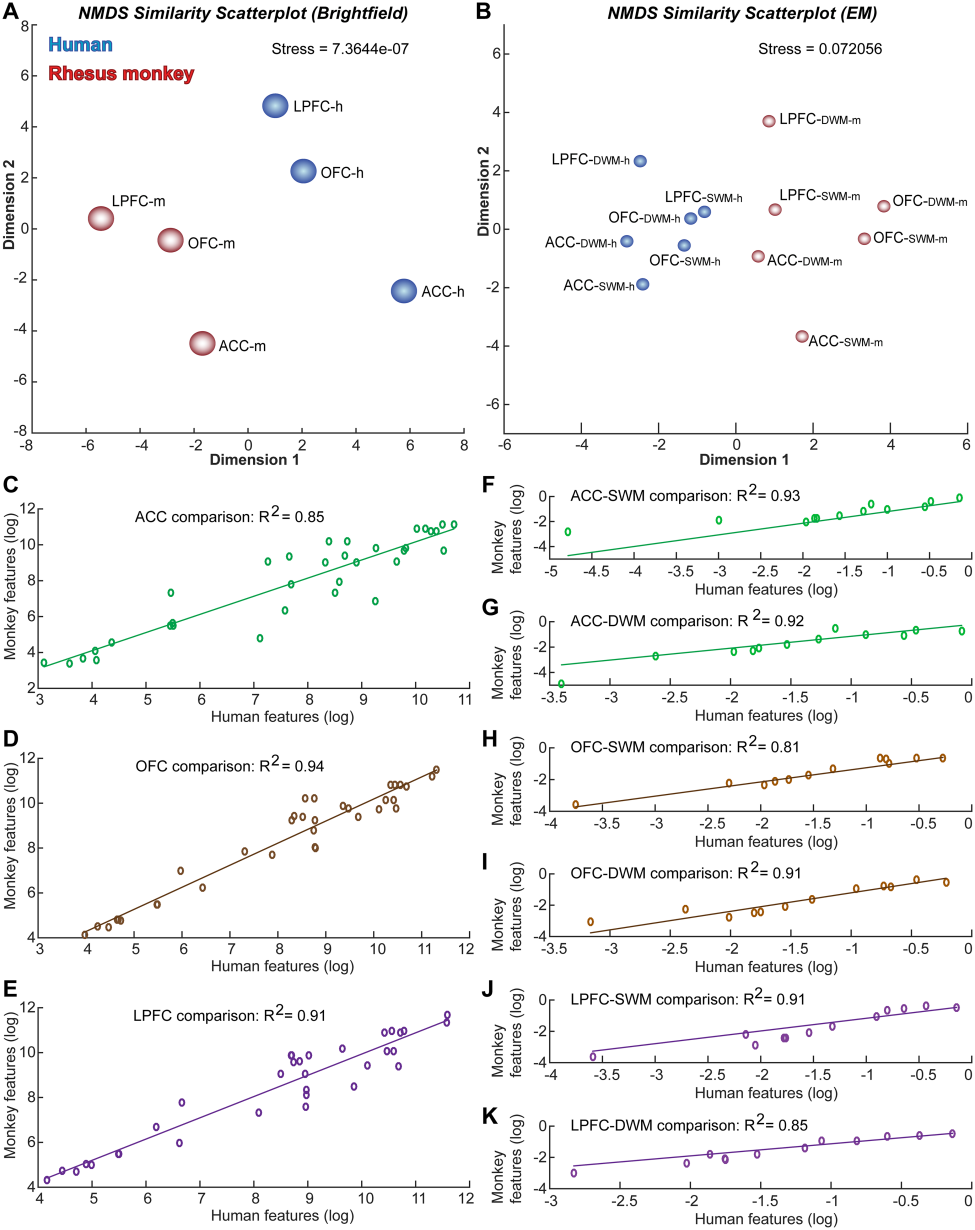
Profile of prefrontal areas based on the gray matter cellular architecture and white matter axon features. (A) Nonmetric multidimensional scaling (NMDS) analysis, based on 34 gray matter architectonic features measured at the light microscope (brightfield), showed a clear separation of 3 prefrontal regions (anterior cingulate cortex [ACC], orbitofrontal cortex [OFC], and lateral prefrontal cortex [LPFC]) in each species (human: h, blue; monkey: m, red) but also revealed a clear separation of the 2 species. Importantly, the same analysis showed that separation of ACC, OFC, and LPFC followed the same pattern across the 2 species. (B) NMDS analysis based on 12 superficial (SWM) and deep (DWM) white matter architectonic features of myelinated axons measured at the electron microscope (EM) showed again a clear separation of 3 prefrontal regions (ACC, OFC, and LPFC) in each species (human: h, blue; monkey: m, red) and a clear separation of the 2 species. Importantly, this analysis also showed that separation of ACC, OFC, and LPFC followed the same pattern across species, with the DWM vector appearing on top of the SWM vector for each area. (C–E) Regression analysis of the log-transformed brightfield feature set for ACC (C), OFC (D), and LPFC (E) showed high correlation between human and rhesus monkey gray matter architecture. (F–K) Regression analysis of the log-transformed EM feature set for ACC (F, G), OFC (H, I), and LPFC (J, K) showed high correlation between human and rhesus monkey SWM and DWM white matter architecture. The numerical data underlying this figure can be found in S1 Data.

To assess interspecies and interareal similarities and differences, we used hierarchical cluster analysis and subsequent multidimensional scaling of 34 features derived from brightfield analysis and 12 feature dimensions from EM analysis (see S1 Data for data features used). This analysis made it possible to project high-dimensional data into a 2-dimensional space. The distance between data points reflects the similarity/dissimilarity of areas. Analyses revealed a clear separation of ACC, OFC, and LPFC areas in each species, based on cellular and axon features of the gray and white cortical matter. The separation of regions was strikingly similar in the 2 species (Fig 10A and 10B). The stress was low, indicating that the low-dimensional representation accurately captured the relationships between areas. The nonmetric multidimensional scaling (NMDS) analysis shows that for both species, there is a gradient from dysgranular to eulaminate cortices. Trends for the monkey and human regions were parallel but separated, indicating that the feature set also reflects species-level differences.

We also examined whether feature sets of each region between species were correlated. Because the estimated variables in the brightfield and EM feature sets of each region included values that varied considerably in scale (e.g., neuron density in tens or hundreds of thousands and myelin optical density from 0 to 255), we log-transformed the data and then regressed the monkey feature set onto the human feature set for each area. The R-squared values were high, indicating high correlation between the monkey and human feature sets for each PFC region for architectonic gray matter data obtained using brightfield microscopy (Fig 10C–10E) and white matter data obtained with EM (Fig 10F–10K).

### White matter axon features reflect PFC connectivity

**Fig 11:**
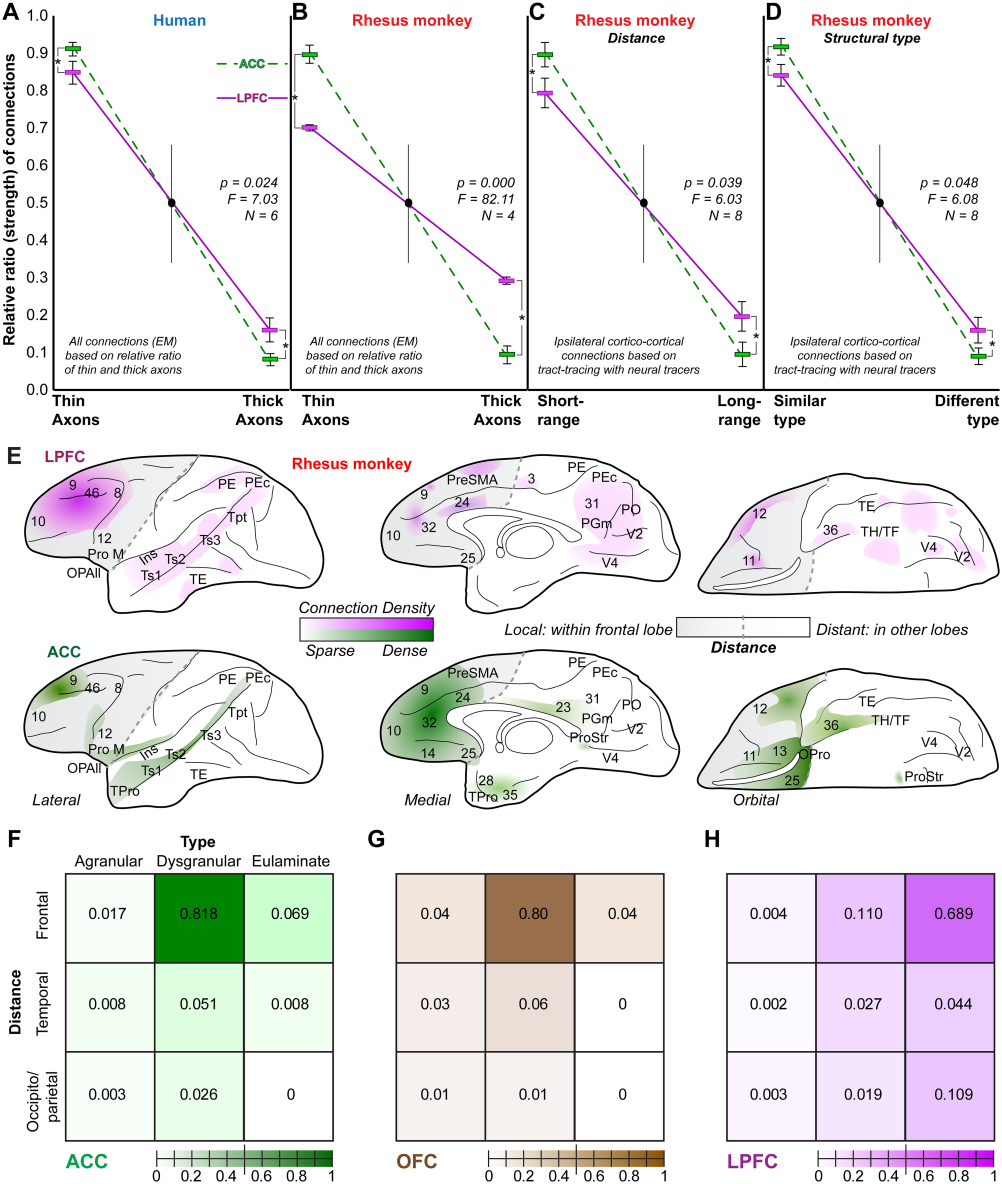
Density of thin and thick axons in the prefrontal cortex (PFC) white matter reflects short-range and long-range connectivity between areas of similar or different structure. (A) Relative ratio of thin to thick axons in human anterior cingulate cortex (ACC) and lateral prefrontal cortex (LPFC) based on the study of axons in the white matter at the electron microscope (EM) (thin/thick axons ± SE in ACC, 92/8 ± 1%; in orbitofrontal cortex [OFC], 86/14 ± 2%, not shown; and in LPFC: 85/15 ± 3%). (B) Relative ratio of thin to thick axons in rhesus macaque ACC and LPFC based on the study of axons in the white matter at the EM (thin/thick axons ± SE in ACC, 89/11 ± 2%; in OFC, 86/14 ± 2%, not shown; and in LPFC: 71/29 ± 0.4%). (C) Relative ratio of short-range to long-range connections in rhesus macaque ACC and LPFC, based on tract-tracing studies of ipsilateral corticocortical connections labeled with neural tracers (short-range/long-range connections ± SE in ACC: 90/10 ± 3%; in OFC, 87/13 ± 4%, not shown; and in LPFC: 80/20 ± 4%). (D) Relative ratio of connections with similar or different types of cortices in rhesus macaque ACC and LPFC, based on tract-tracing studies of ipsilateral corticocortical connections labeled with neural tracers (similar/different cortical type connections ± SE in ACC, 92/8 ± 2.4%; in OFC, 87/13 ± 1.6%, not shown; and in LPFC: 84/16 ± 3%). (E) Lateral (left), medial (center), and orbital (right) surface heat maps of the rhesus macaque brain show the relative strength of connections of LPFC (top) and ACC (bottom) with other nearby (frontal) or distant (temporal, parietal, and occipital) cortices. The maps are based on extensive quantitative data from tract-tracing studies with neural tracers. The frontal lobe that includes short/medium-range pathways is shaded in light gray and is separated from other lobes with a dotted line. (F–H) Matrices of the relative strength of the connections of ACC (F), OFC (G), and LPFC (H) with nearby or distant cortical areas of similar or different type. Frontal areas are grouped as nearby, and temporal and occipito/parietal areas are grouped as distant. We used 3 structural types for this analysis: agranular (no layer 4), dysgranular (thin layer 4), and eulaminate (6 well-delineated layers, thick layer 4). Analyses showed that all PFC cortices are primarily connected with nearby cortices of similar structural type. LPFC had relatively more connections with distant cortices of similar type compared to ACC. The tract-tracing connectional data paralleled the EM data from the study of the thickness and density of white matter axons below the areas studied and suggested that in monkeys and humans thin axons primarily link nearby areas of similar structural type through robust connections, while thick axons participate in long-distance connections. ACC, shades of green and dotted lines; OFC, shades of brown; and LPFC, shades of purple and purple solid lines. Asterisks indicate significant differences (1-way ANOVA). Abbreviations: Ins, Insula; OPAll, orbital periallocortex; OPro, orbital proisocortex; Pro M, motor proisocortex; PE, posterior parietal area PE; PEc, posterior parietal area PEc; PGm, posterior parietal area PGm; PO, parietoccipital area; PreSMA, pre supplementary motor area; ProStr, area prostriata; TE, inferior temporal area; TH/TF, parahippocampal areas; TPro, temporal proisocortex; Tpt, temporoparietal area; Ts1, superior temporal area 1; Ts2, superior temporal area 2; Ts3, superior temporal area 3; V2, visual area 2; V4, visual area 4. The numerical data underlying this figure can be found in S1 Data.

The above analysis established that rhesus monkeys and humans show similar trends in the architecture of axons below distinct prefrontal regions. We then investigated whether axon features can be used to infer connectivity. This was accomplished by comparing the high-resolution EM data on the relative ratio of thin and thick white matter axons in humans and monkeys with connectivity data for ACC, OFC, and LPFC areas in monkeys. Monosynaptic connections were studied at high resolution using tract tracing in monkeys. We used available data on projection neurons throughout the cortex that are directed to ACC, OFC, and LPFC areas in rhesus monkeys to compare directly with axon features. We expressed labeled projection neurons as the relative ratio of short/medium-range versus long-range cortical connections (*N* = 11 animals, 12 tracer injections; 6, female), based on the distances of interconnected areas. Short/medium-range cortical connections of ACC, OFC, and LPFC were restricted within the frontal lobe; long-range axons linked prefrontal areas with temporal, parietal, and occipital cortices; the results are shown in Fig 11. All areas had overall more local (within the frontal lobe) than distant connections. However, the relative proportion of short-range versus long-range connections for each region was significantly different such that LPFC made more long-range connections than ACC (Fig 11C and 11E). As in the other analyses (above), OFC showed intermediate features with values between the other 2 PFC regions (not shown). These relative ratios closely resembled the EM data that included all axons in the white matter below ACC, OFC, and LPFC, placing thin axons in short/medium-range pathways and thick axons in long-range pathways (Fig 11B and 11C).

Our findings of systematic trends in the architecture of the cortex confirm and extend classical architectonic studies and, importantly, provide novel findings on the architecture of the white matter beneath the cortex. An important principle is that the architectonic differences in the cortex are systematic and connections are predicated on the relationship of the laminar structure (cortical type) of the linked areas, according to the structural model for connections (reviewed in [21]). Moreover, the principle of the relationship of cortical type and connections transcends the model of cortical connectivity based on the distance between areas [4, 72], as illustrated by the fact that some distant areas of similar cortical type are strongly interconnected [50, 73–75]. We thus expressed the relative number of labeled projection neurons from the above analysis as the relative ratio of connections of areas with similar or different structural types. We grouped cortical areas into 3 major, well-established structural types that are widely and consistently used in the literature: agranular, dysgranular, and eulaminate (for reviews, see [76, 77]). Results showed that the limbic ACC and posterior OFC areas were primarily connected with other dysgranular cortices, whereas LPFC areas were primarily connected with other eulaminate areas (Fig 11D). The relative ratios of connections grouped by structural type closely resembled the EM data that included all axons in the white matter below PFC areas, placing thinner axons in pathways that connect mostly neighboring areas of similar type (Fig 11B and 11D); this finding is consistent with the principle of systematic variation of areas across the cortical mantle (reviewed in [21]).

The relationship of thin and thick axons in the white matter of human PFC showed the same trend as in monkeys, with overwhelming predominance of thin compared to thick axons. As was the case in rhesus macaques, human ACC had relatively more thin axons compared to LPFC (Fig 11A), while OFC showed intermediate levels.

We then used the rich tract-tracing connectivity database in rhesus macaques to quantitatively map the strength of ACC, OFC, and LPFC connections in relation to the distance between linked areas, in order to identify short- and long-distance connections, and classify them also by the structural type of interconnected cortices (Fig 11E–11H). This analysis revealed that areas within all 3 PFC regions were primarily connected with nearby cortices of similar type. In addition, LPFC, in particular, had relatively more distant connections with areas of similar type, compared to ACC.

### Axon features differentiate pathways in ASD compared to control human brains

**Fig 12:**
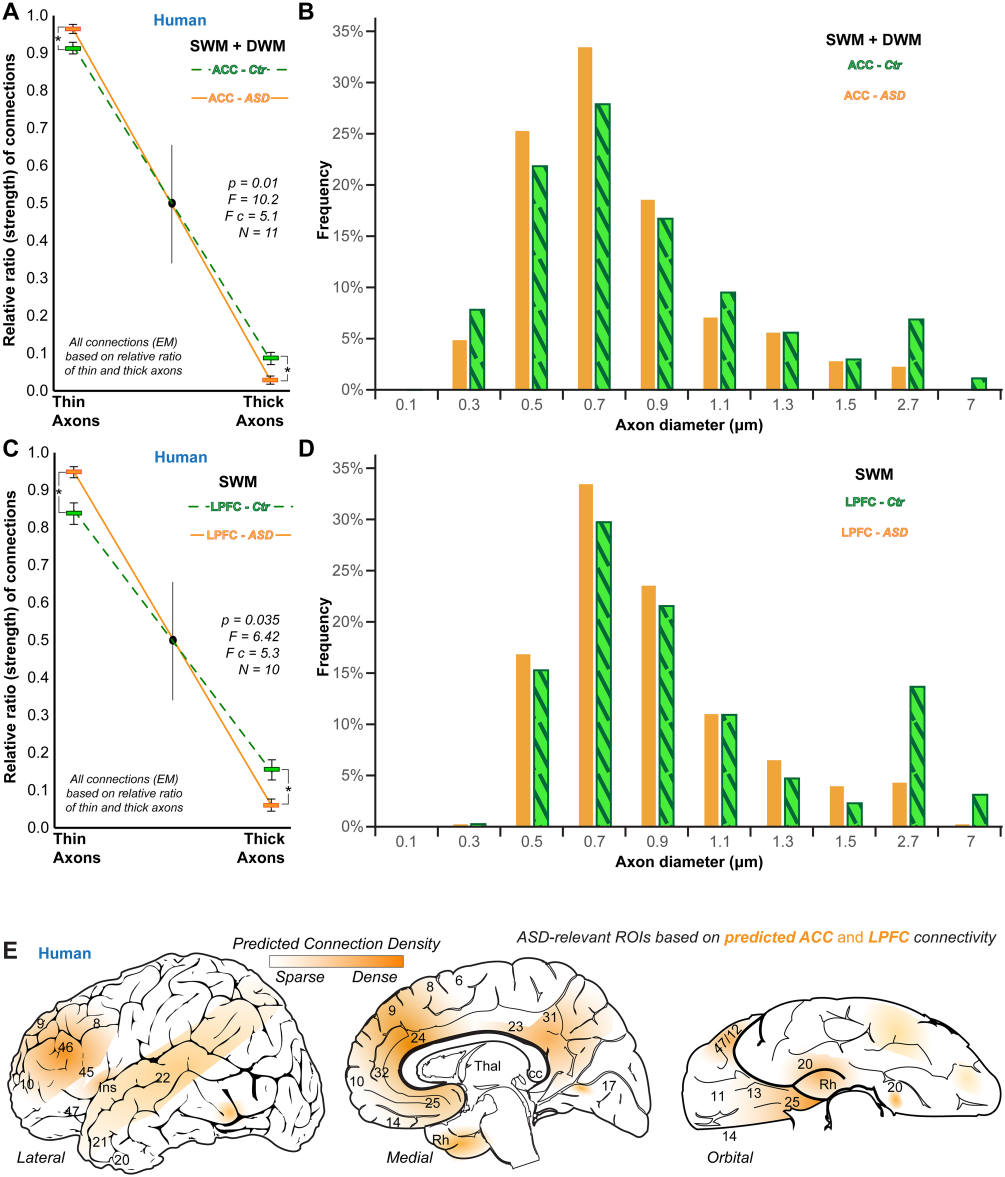
Changes in axons below the anterior cingulate cortex (ACC) and the lateral prefrontal cortex (LPFC) in autism spectrum disorder (ASD) point to a disruption of key prefrontal circuits and networks. (A, B) Superficial white matter (SWM) and deep white matter (DWM). (A) White matter below the ACC in individuals with ASD (orange) had significantly more thin axons and fewer thick axons compared to neurotypical adults (Ctr, green; ratio of thin/thick axons ± SE in Ctr, 92/8 ± 1%; in ASD, 98/2 ± 0.5%). The changes were significant in the entire white matter (SWM and DWM). (B) Frequency distribution of axon sizes in the white matter below the ACC from all control (Ctr, green, stripes) and all ASD (orange) subjects combined also shows the exuberance of thin axons (≤1.5 μm) and the reduction of thick axons (>1.5 μm) in ASD. (C, D) SWM only: thin/thick axon ratio (C) and frequency distribution (D) of axon sizes in SWM below LPFC parallel changes in ACC, with more thin and fewer thick axons in ASD (orange) compared to control (green) subjects (ratio of thin/thick axons ± SE in Ctr, 85/15 ± 3%; in ASD, 95/5 ± 1%). (E) Lateral (left), medial (center), and orbital (right) surface heat maps of the human brain show cortical regions likely affected in ASD based on predicted ACC and LPFC connectivity and predicted relative strength of connections with other nearby (frontal) or distant (temporal, parietal, and occipital) cortices. Numbers indicate Brodmann’s areas. Abbreviations: cc, corpus callosum; EM, electron microscope; Ins, insula; Rh, rhinal cortices; ROI, region of interest; Thal, thalamus. Asterisks in panels A and C indicate significant differences (1-way ANOVA, *N* = 11 subjects; 5 ASD subjects). The numerical data underlying this figure can be found in S1 Data.

The findings in ACC were of particular interest in view of our previous data, which showed that individuals with ASD had significantly more thin axons in the SWM and fewer thick axons in the DWM below ACC [64]. We used the expanded dataset of neurotypical-control subjects in this study (*N*_CTR_ = 6, Table 3 shows subjects used in EM study of white matter), which contained axons from the portion of area 32 anterior to the corpus callosum that precisely matched the ACC white matter region studied earlier [64], and compared axon features in the brain of adults with ASD (*N*_ASD_ = 5, Table 3). For this analysis we used the outer diameter of axons, which takes into account the myelin sheath, and parcellated the population of thin and thick axons based on the cluster analysis from the expanded control dataset. In agreement with our previous report, the brains from adults with ASD had significantly more thin axons and fewer thick axons in the white matter below ACC area 32 (Fig 12A). The addition of control subjects in the expanded dataset increased the power of the analysis and showed that the differences between control and ASD subjects were pronounced. Moreover, the changes in white matter axons in ASD could be reliably detected within the entire population of axons and throughout the entire white matter below ACC (Fig 12A and 12B).

Further, the extended dataset of control subjects additionally revealed, for the first time (to our knowledge), similar changes in the SWM below dorsal LPFC area 46. Thus, area 46 also had significantly more thin axons and fewer thick axons in adults with ASD compared to neurotypical controls (Fig 12C and 12D).

It should be noted that some of the additional control subjects we used in this study were older (58–67 years) than other control and ASD subjects (30–50 years, Table 1). This is important because structural changes in the white matter with aging have been reported—in particular, decreases in axon density and the thickness of the myelin sheath in major pathways of the monkey (reviewed in [78, 79]) and human cortex (reviewed in [80–83]). To assess potential effects of age on estimates of axon size and density, we examined the correlation of all estimated variables between subjects within control and ASD groups, using multivariate analysis of covariance (MANCOVA) with age as a covariate. Age had no effect on the results. Estimated variables from older control subjects were well within the range of values from younger control subjects (see S1 Data).

## Discussion

Our findings provide direct evidence that the gray and white matter of the human and rhesus monkey PFC vary systematically and in parallel. This novel finding allowed study of short- and long-range connections between structurally similar and dissimilar cortices in rhesus monkeys. The high-resolution data on the gray and white matter architecture and the direct relationship to connections establish a template to study pathways in humans and their disruption in disease, as revealed here for ASD.

### Architectonic differences reflect enlargement of the human PFC compared to monkeys

The organization of the PFC appears to be largely preserved in primate evolution, rendering nonhuman primates an invaluable animal-model to study connections in humans [1, 17, 19, 28–33, 84]. The frontal lobe, in particular, has expanded significantly in humans compared to nonhuman primates [1, 31, 85–89], accompanied by more and larger neurons, more synapses, and greater complexity in networks [84, 90–95]. Indeed, a notable architectonic difference in PFC between the 2 primate species was the lower neuron density found in the human PFC, leaving more space available for neuronal processes and synapses [45]. Our analyses suggest that the human PFC and, in particular, its limbic components are endowed with high plasticity as well as vulnerability in neurological and psychiatric diseases [96–98].

The human PFC white matter had overall thicker axons than in monkeys, consistent with the longer distances that pathways must traverse in a larger brain, as also observed in other comparative studies [1, 65, 89]. The overall enlargement of axons and the thickness of the myelin sheath in human PFC is consistent with the increased density of oligodendroglia [32, 99, 100] and helps explain the increase in the glia/neuron ratio in human cortex compared to nonhuman primates [90]. These findings thus reveal that in addition to gray matter architecture [5, 24, 27, 34, 53, 60, 64], we can use white matter features to construct unique fingerprints of cortical areas and facilitate their distinction in primates.

### Variation in gray and white matter structure follows similar trends in monkeys and humans

The relationships among the 3 PFC regions in humans and monkeys followed remarkably similar patterns. Thus, overall density of neurons, oligodendrocytes, and intracortical myelin increased progressively from the limbic ACC and OFC to eulaminate LPFC areas [34, 35, 45, 64, 90, 96, 101]. The gradual increase in laminar elaboration and density of neurons from limbic to eulaminate PFC areas was marked by a parallel increase in the density of myelinated axons in the white matter [32]. In both species, the ACC and the OFC, 2 limbic regions, had relatively more thin and fewer thick axons compared to the LPFC. Parallel analysis of the rhesus macaque connectome showed that ACC and OFC also had denser short/medium-range pathways compared to LPFC, which also had relatively dense long-distance connections. Myelination can accelerate impulse conduction and regulate the timing of communication among local or distant connections [62] and the synchronization of functionally related areas [65, 102–104]. Our findings are thus consistent with a role of limbic ACC areas in networks that process signals at relatively slow speeds, such as pain [105, 106]. On the other hand, LPFC areas maintain strong connections with select distant sensory and other association areas implicated in cognitive functions.

### Gray and white matter architecture reflects PFC connectivity in primates

Our findings thus showed a similar architecture in the gray and white matter of human and rhesus monkey ACC, OFC, and LPFC. Moreover, differences in the architecture of the gray and white matter varied systematically and in the same direction in both species. In addition, the gray and white matter architecture accurately reflected the connectivity in macaque monkeys and, by inference, in humans.

These findings are rooted in the general principle of systematic variation in the cortex. This principle is based on strong evidence that architectonic variations in the cortex are not random but systematic [45]. Further, corticocortical connections mirror the systematic architectonic variation, as formulated in the “structural model for connections” [107, 108]. According to this model, connections between areas are biased toward a “feedforward” or a “feedback” pattern depending on the (dis)similarities of the architecture between linked areas. The bigger the architectonic differences, the bigger the bias. For example, dysgranular limbic areas, like those of the ACC and the posterior OFC, which have less delineated layers and lower cell density, send mainly feedback projections to eulaminate areas, like those of the LPFC, which have 6 well-defined layers and higher neuron density. These projections originate mainly from the deep layers of ACC or OFC and terminate mostly in the superficial layers of the LPFC [38, 108, 109]. Projections in the opposite direction, from LPFC to ACC, are feedforward: they originate mostly from the superficial layers and terminate in the middle/deep layers of ACC [38, 108]. Connections between areas with similar laminar structure are columnar: they originate in most layers and terminate in all layers, as seen in the connections linking dysgranular ACC and OFC areas [56]. Over the past 20 years, studies have consistently supported this model for ipsilateral and callosal connections among diverse cortices in nonhuman primates (e.g., [57, 73, 75, 107, 108, 110–112]) and other species [113, 114].

The relative distribution of neurons in different layers in areas that differ in laminar structure (cortical type) is consistent with their predominant projection pattern. For example, the limbic ACC areas have relatively higher density of neurons in the deep layers, their main output layers, and neurons in layer 5 of ACC are bigger than in other layers. In contrast, eulaminate LPFC areas have denser layers 2 and 3, and their biggest neurons are found in layer 3, their predominant output layer to other cortices [107, 108].

The present findings further showed that the principle of systematic variation is also embedded in the fine structure and density of myelinated axons in the white matter. The white matter below PFC thus contained mostly thin myelinated axons and few thick axons, consistent with their connections being primarily with neighboring frontal areas [1, 64, 65, 115], in line with previous studies [65, 116]. This finding is also consistent with the observation that nearby areas tend to be strongly interconnected, forming “rich club” hubs that attract and disperse communication paths [67, 117].

Further, our results revealed that the relative ratio of thin to thick axons in the white matter below primate PFC (and by extension connections) is associated with a more fundamental principle than proximity—namely, cortical type. This principle most parsimoniously explains why both nearby as well as distant areas can be strongly connected [50, 57, 73–75, 108, 118]. Consequently, areas that are robustly interconnected, whether they are neighbors or not, are more likely to have comparable overall laminar structure. By probing the organization of the components of the white matter, our findings now also differentiate between short/medium-range from long-distance pathways. Short/medium-range pathways connect nearby areas, as verified in the connections in monkeys and supported by the prevalence of thin myelinated axons in the SWM. On the other hand, long-range pathways that must travel in the DWM contain thicker axons, as shown by the preponderance of thick axons below LPFC, in accord with its strong connections with distant parietal, temporal, and occipital areas. These findings are in line with recent imaging studies that use MRI and diffusion-weighted tractography methods to map the human and monkey cortex based on the relationship of whole-brain white matter connectivity with macroscopic structural features, like T1- and T2-based intracortical myelin content, or the correlated activity of areas during rest, unimodal, and transmodal functions [3, 5, 24, 119–121].

### White matter axon features reveal disrupted neural pathways in ASD

The regularity and similarity in the structure of the gray and white matter in the PFC of primates suggests that architectonic information can be used to predict the strength and laminar pattern of connections in humans. While invasive procedures for the detailed study of connections are precluded in humans, the architecture can be studied in postmortem brain tissue [63, 64], as in this study. Based on the similarities and functional data for ACC, OFC, and LPFC areas [122–124], we predict that these cortical regions will have a similar pattern of connections in humans as in rhesus macaques [39, 56, 107, 109], which awaits further test in future studies.

Our findings have significant implications for the status of the architecture and, consequently, for connections in neurological and psychiatric disorders and suggest that parallel gray and white matter changes may reflect common pathological mechanisms, in line with other imaging and neuropathological studies [63, 125–128]. The PFC, in particular, is consistently affected in ASD, along with the processes of attention, social interactions, emotions, and executive control [36, 97, 129–132]. Prefrontal pathways are structurally and functionally disorganized in autism, exhibiting local overconnectivity and long-distance disconnection [63, 64, 133–141]. Our findings of more thin than thick axons in the PFC of individuals with ASD support the hypothesis that PFC is “talking to itself” in autism [132]. The disruption appears to be particularly extensive below ACC, with an exuberance of thin axons (typically found in short-range pathways) and a decrease in thick axons (typically found in long-range pathways).

Our analyses of the architectonic profile of the primate PFC and connectome delineate the preponderant pathways linking PFC with other areas (Fig 12E). In the case of dysgranular ACC areas, these networks include strong columnar connections with nearby medial PFC and OFC, as well as relatively weaker long-range connections with similarly dysgranular rhinal, temporal, posterior cingulate, and prostriate visual cortices [14, 50, 56, 73–75, 111, 142, 143]. In the case of eulaminate LPFC, these networks include strong columnar connections with nearby PFC, as well as moderate/strong long-range connections with eulaminate lateral parietal, superior temporal, and occipital cortices [57, 73, 144].

On the other hand, in connections between structurally dissimilar cortices, our results highlight the potential significance of robust short-range connections between dysgranular ACC and eulaminate LPFC areas [38, 145, 146]. In rhesus macaques, for example, excitatory axons from ACC send feedback pathways that mostly target the superficial layers of LPFC. When these pathways form synapses with inhibitory neurons in LPFC, they innervate preferentially inhibitory neurons that have modulatory effects on pyramidal neurons [109] and likely increase the signal-to-noise ratio and facilitate focused attention on a task [147]. The exuberance of thin axons in the white matter below ACC and the SWM below LPFC in ASD could underlie behavioral challenges typical of autism, like excessive focus and inability to shift attention when necessary. Our findings support a potential disruption of mechanisms that rely on a fine balance of excitation and inhibition in the PFC and are consistent with atypical ACC and LPFC activation in ASD [135, 148, 149], including desynchronized and reduced activity during working memory tasks ([137, 150]; reviewed in [151]).

In conclusion, our findings provide the basis to relate data from invasive neuroanatomical tract-tracing studies in a nonhuman primate model, the rhesus monkey, to neuroanatomical, histopathological, or noninvasive imaging studies in humans. This analysis is a prerequisite for understanding brain function and disruption in neuropathology and for identification of potential regions for intervention in disease. Our approach establishes a framework of structural principles based on detailed high-resolution quantitative findings that complement a large body of data on the cortical architecture in human [35, 45, 64] and nonhuman primates [34, 38, 107, 108] and connections of prefrontal areas [14, 57, 75, 107, 108, 110]. Application of these principles makes it possible to study and model functionally relevant cortical circuits at an exquisite level of refinement in humans, as exemplified by the study of prefrontal networks, and their consistent disruption in disorders like ASD.

## Materials and methods

### Experimental design

We used prefrontal postmortem brain tissue from neurotypical adult humans (*N* = 8, female: 3) and rhesus monkeys (*Macaca mulatta*; *N* = 25, female: 12; Tables 1–3). We quantitatively studied the cyto- and myeloarchitecture of the gray and white matter of ACC (areas 25 and 32), OFC (areas 13 and OPro), and LPFC (areas 46 and 8), at high resolution, using brightfield and electron microscopy (Fig 1). We then quantitatively characterized cellular and axonal structures and densities to distinguish PFC areas within and across species. We used these data to compare and correlate structural features of short- and long-range pathways between areas of similar or different structure with connectivity data from quantitative tract-tracing studies in rhesus macaques. Finally, using this framework that links the structure of cortical areas with their connections, we compared PFC white matter organization and connectivity between neurotypical adults (*N* = 6, female: 3) and individuals with ASD (*N* = 5, female: 1), to study short- and long-range ACC and LPFC pathways with structurally similar or different cortices. Table 3 shows the human subjects and rhesus monkeys used in each type of experiment and analysis. Data of individual human subjects and rhesus monkeys are included in the Supporting Information (S1 Data).

### Human postmortem brain and tissue preparation

We used coronal PFC sections from formalin-fixed postmortem brain tissue of neurotypical adults and individuals with ASD. Brain tissue was obtained from the Harvard Brain Tissue Resource Center through the Autism Tissue Program and Anatomy Gifts Registry. The study was approved by the Institutional Review Board of Boston University. Human subjects were matched as closely as possible based on tissue availability. Brain tissue from all 8 neurotypical (control) subjects was processed and used for quantitative light microscopic analysis. Brain tissue from 6 of these subjects (3 female subjects) was processed and used for quantitative electron microscopic analysis (Table 3). Two control subjects (HBJ and HBK) were not used for EM because of suboptimal tissue quality. The diagnosis of autism was based on the Autism Diagnostic Interview–Revised (ADI-R). Some subjects from individuals with ASD were diagnosed with seizure disorder (subject AN 08792), depression (subject AN 18892), and schizophrenia (subject AN 06746). Results from the analysis of the features of axons in these and the female subjects (HAW, B-5353, B-6004, and AN-07770) did not differ from other subjects within each group, in this and other studies that used tissue from the same subjects [64, 152, 153]. Clinical characteristics, including ADI-R scores, and other data of human subjects and the experiments they were used for are summarized in Tables 1 and 3 and S1 Table.

Coronal PFC blocks were matched (Fig 1A) based on human brain atlases [44, 154] and additional cytoarchitectonic studies of human PFC [35, 45, 59]. We postfixed tissue in 2% paraformaldehyde and 2.5% glutaraldehyde, in 0.1 M phosphate buffer (PB, pH: 7.4) for 2–4 days at 4 °C. To preserve the ultrastructure until processing, tissue blocks were cryoprotected in 25% sucrose solution and then immersed in antifreeze solution (30% ethylene glycol, 30% glycerol, 40% 0.05 M PB, pH: 7.4 with 0.05% azide) and stored at −20 °C. Tissue blocks were then rinsed in 0.1 M PB and cut coronally in 50-μm-thick sections on a vibratome (Pelco, series 1000) or frozen in −70 °C isopentane and cut in a cryostat (CM 1500, Leica) in the coronal plane at 20–50 μm in 10 series of free-floating sections. Sections used for histological stains were mounted on chrome-alum gelatin–coated slides.

### Animals, surgery, tracer injections, and tissue preparation

We used archival postmortem rhesus monkey (*M*. *mulatta*) brain tissue for architectonic and pathway analyses (Tables 2 and 3 and S2 Table). We used 12 tracer injections and data plots from 11 rhesus monkeys to study the cortical connections of ACC (*N* = 4), OFC (*N* = 4), and LPFC (*N* = 4) through tract tracing. Tracer injection sites and quantification of projection neurons have been described in detail in previous studies and will be briefly described here ([14, 56, 57, 73, 75, 108–112, 143, 155–161]; see S2 Table for a detailed list of relevant quantitative studies). The available rhesus monkeys were young adult animals, obtained from the New England Primate Research Center. Procedures were designed to minimize animal suffering and reduce the number of animals used. Detailed protocols of the procedures were approved by the Institutional Animal Care and Use Committee at Harvard Medical School and Boston University School of Medicine in accordance with NIH guidelines (DHEW Publication no. [NIH] 80–22, revised 1996, Office of Science and Health Reports, DRR/NIH, Bethesda, Maryland, United States). Table 3 summarizes the data of rhesus monkeys and their specific use here.

Imaging for injection of tracers, surgery, perfusion, and tissue preparation were described previously [13, 14, 16]. The retrograde tracers injected in the ACC, the OFC, and the LPFC included biotinylated dextran amine (BDA, 10% solution, Invitrogen, Carlsbad, California, US), Fast Blue (FB, 1% solution; Sigma, St. Louis, Missouri, US), Diamidino Yellow (DY, 3% solution; Sigma), and horseradish peroxidase conjugated to wheat germ agglutinin (HRP-WGA, 8% solution, Sigma). After removal from the skull, all brains were photographed, cryoprotected in a series of sucrose solutions (10%–30% in 0.01 M PBS), and frozen in −70 °C isopentane (Fisher Scientific, Pittsburg, Pennsylvania, US) for rapid and uniform freezing. Brains were cut in the coronal plane on a freezing microtome at 40 or 50 μm to produce 10 matched series. In animals with injection of fluorescent tracers, 1 series was mounted on glass slides, coverslipped, and used to map labeled neurons.

### Nissl and Gallyas staining for optical microscopy

Series of sections of monkey and human PFC were mounted on gelatin-coated slides (Gelatin Type A, G8-500, Fisher Scientific, Fair Lawn, New Jersey, US) and stained for Nissl using thionin blue (Thionin powder, T-409, Fisher Chemicals) to view neurons and glia and examine the cytoarchitecture of each area, as described [162]. Briefly, sections were dried, defatted in a 1:1 solution of chloroform (C298-1, Fisher Scientific) and 100% ethanol (Pharmco-AAPER, Brookfield, Connecticut, US) for 1 to 3 hours, rehydrated through a series of graded alcohols and dH_2_O, stained with 0.05% thionin (pH 4.5) for 15 minutes, differentiated through graded alcohols, cleared with xylenes (UN1307, Fischer Scientific), and coverslipped with Entellan (Merck, Whitehouse, New Jersey, US). Other series of sections mounted on gelatin-coated slides were stained using the Gallyas silver technique to label intracortical myelin [163, 164].

### Electron microscopy

We postfixed human and monkey tissue blocks or sections in 2%–4% paraformaldehyde and 2.5%–3% glutaraldehyde, in 0.1 M PB, pH 7.4, for 2 days at 4 °C. To preserve the ultrastructure until processing, tissue blocks and sections were immersed in antifreeze solution (30% ethylene glycol, 30% glycerol, 40% 0.05 m PB, pH 7.4, with 0.05% azide) and stored at −20 °C. Blocks were rinsed in 0.1 M PB and cut coronally in 50-μm-thick sections on a vibratome (series 1000, Pelco) or on a freezing microtome. Sections were rinsed in 0.1 M PB and postfixed in a variable wattage microwave oven (Biowave, Pelco) with 6% glutaraldehyde at 150 W. Small regions of sections containing the superficial or deep parts of the white matter below the PFC were cut under a dissecting microscope. We confirmed the presence of ACC, OFC, and LPFC cortical regions of interest in adjacent sections that were stained with Nissl. White matter regions of interest were postfixed in 1% osmium tetroxide with 1.5% potassium ferrocyanide in PB, washed in buffer (PB) and water, and dehydrated in an ascending series of alcohols. While in 70% alcohol, they were stained with 1% uranyl acetate for 30 minutes. Tissue sections were then cleared in propylene oxide and embedded in araldite or LX112 at 60 °C. Serial ultrathin sections (50 nm) were cut in the horizontal plane with a diamond knife (Diatome, Fort Washington, Pennsylvania, US) using an ultramicrotome (Ultracut; Leica, Wein, Austria) and collected on single slot grids to view with a transmission EM (100CX; Jeol, Peabody, Massachusetts, US) or a scanning EM (Zeiss Gemini 300 with STEM detector and Atlas 5 software modules), as described [64, 162]. Myelinated axons were easily identified at the EM by the darkly stained electron-dense myelin sheath [165].

### White matter segmentation

We subdivided white matter beneath ACC, OFC and LPFC in 2 regions, by determining axon alignment in serial sections under the microscope, at gradually increasing distances from the gray-white matter border, as described [63, 64]. The outer, gyral, or superficial part (SWM), which mainly consists of axons that participate in short-range pathways, was immediately adjacent to layer 6 of the overlying cortical areas. The SWM, which was segmented based on the predominant radial alignment of axons, had a thickness of 0.5–2.5 mm. However, some axons in the SWM either had variable trajectories or followed the curvature of the overlying gray matter, resembling the likely trajectory of U-shaped fibers that connect neighboring gyri [28, 70]. The inner or deep part of the white matter (DWM), which mainly consists of axons participating in long-distance pathways, was segmented based on the predominance of axons that run mainly sagittally to the cerebral surface.

### Sample size

To estimate the sample size, we took into account the number of human subjects and rhesus monkeys and the volume fraction of areas sampled so that the number of individual cells and axons examined produced estimates with a small coefficient of error (<10%), as described [16, 64, 96, 163]. Pilot studies with exhaustive sampling, progressive means analysis, and the formula of West et al., [166], as well as a posteriori power analysis, using data from our previous studies of cell and axon densities, or tract tracing of pathways, took into consideration all known and estimated variables, including age, sex, postmortem interval (PMI), and other diagnoses. These considerations showed that the sampling ratios used exceeded the samples needed to detect differences with a greater than 90% probability and with an estimated large effect size in the population (0.80).

### Unbiased estimate of neurons, oligodendrocytes, and myelinated axons

We estimated the overall and laminar density of neurons and oligodendrocytes in representative columns along the depth of a straight portion of the gyral part of anterior cingulate areas 25 and 32, orbital areas 13 and OPro, and lateral area 46 (Fig 1), based on previous maps for rhesus macaques [38] and humans [44, 59, 154]. We examined OFC area 13 in human PFC and the structurally similar OFC area OPro in rhesus macaques. We used the unbiased stereological method of the optical fractionator [167, 168] with the aid of commercial software (StereoInvestigator; Microbrightfield, Williston, Vermont, US), as described [64, 96, 163]. We used a minimum of 3 sections from 1 series of coronal sections from each human subject and rhesus monkey and drew contours of layers in each column.

**Fig 13:**
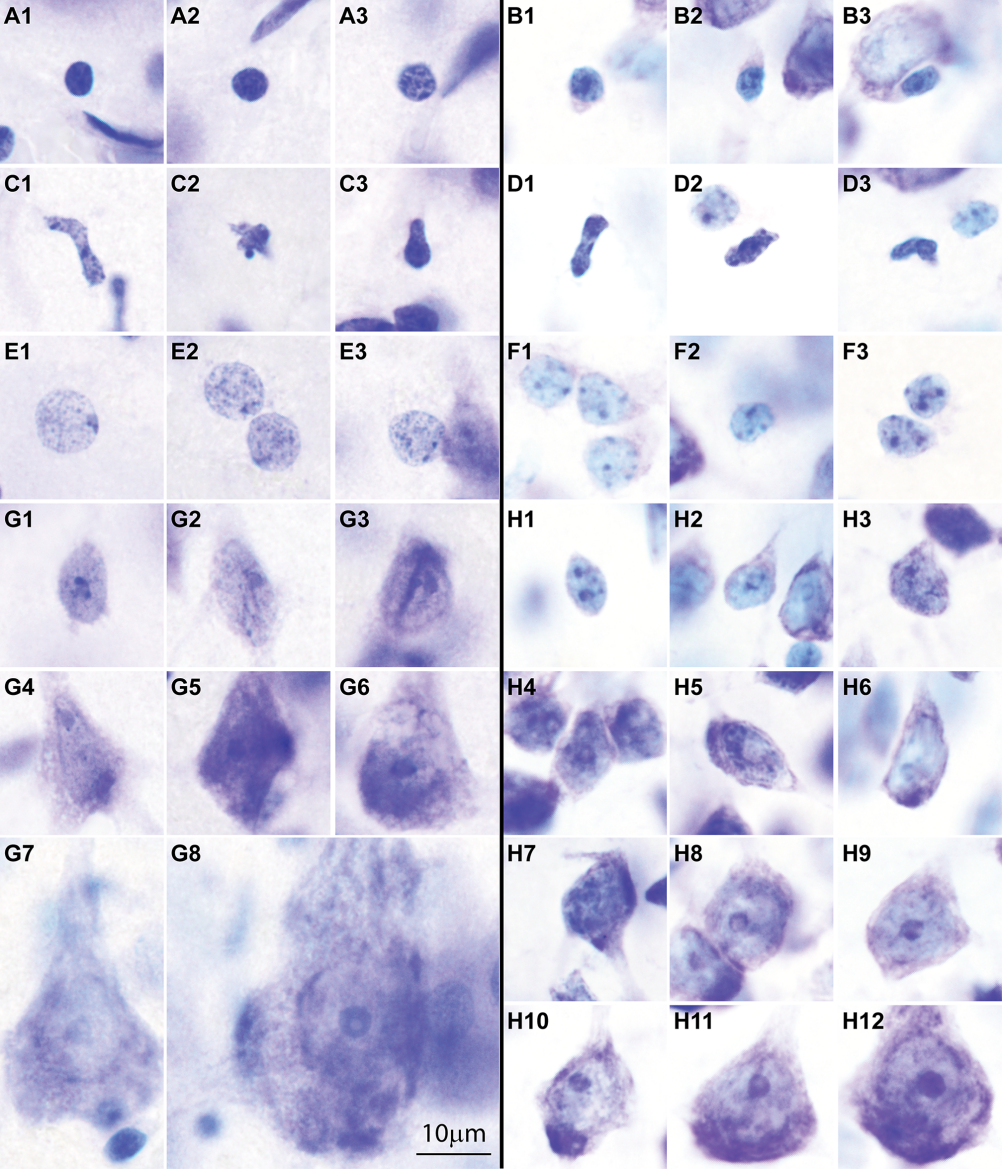
Cytological figures of neurons and glial cell types in Nissl-stained sections of the primate cerebral cortex. (A1–A3) Human oligodendrocytes. (B1–B3) Monkey oligodendrocytes. The nuclei of oligodendrocytes are rounded and darkly stained, with 2–4 darker heterochromatin grains. In the human, the nucleus of some oligodendrocytes appears lighter (A3). (C1–C3) Human microglial cells. (D1–D3) Monkey microglial cells. The nuclei of microglial cells are darkly stained and show a variety of irregular shapes. (E1–E3) Human astrocytes. (F1–F3) Monkey astrocytes. The nuclei of astrocytes are rounded and lightly stained with a rim of heterochromatin under the nuclear envelope and several heterochromatin granules attached to this rim or in the heterochromatin net. The cytoplasm of astrocytes is usually unstained, but some show yellow inclusions and pinkish threads in the perinuclear cytoplasm (F1). (G1–G8) Human neurons. (H1–H12) Monkey neurons. The cytoplasm of neurons is stained in Nissl sections around the entire perimeter of the nucleus. The nucleolus of small neurons is partially or totally surrounded by irregular clumps of heterochromatin, and 1–2 additional heterochromatin granules can be seen in the nucleoplasm (e.g., G1; H1–H3). The nucleus of large neurons looks “empty,” with a distinct nucleolus flanked by small granules of heterochromatin (e.g., G6–G8; H9–H12). Nuclear folding, present in neurons, additionally helped distinguish small neurons from astrocytes, especially in humans (e.g., G3). The calibration bar in G8 applies to A1–H12. For a more detailed description of cytological features of cells in the human and rhesus monkey brain, see [162].

We counted Nissl-stained neurons or oligodendrocytes at 1000×, using systematic random sampling. We identified neurons and oligodendrocytes based on their characteristic features, following a detailed cytology algorithm, as we have described [[162]; Fig 13]. Briefly, we first split labeled cell profiles into 2 broad groups. One group included cells with darkly stained nuclei (microglia and oligodendrocytes), and the other group included cells with a lighter nuclear stain (neurons, astrocytes, and endothelial cells). The level of stain tends to be correlated with the size of the nucleus, that is, darkly stained nuclei tend to be smaller than lightly stained nuclei. Once a cell was allocated into 1 of these 2 broad groups, we followed the detailed neurocytology algorithm [162] to distinguish microglia and oligodendrocytes in the darkly stained nucleus group and neurons, astrocytes, and endothelial cells in the other group, using key cytological features. These included the presence or absence of cytoplasm around the nucleus (present in neurons; absent in glial cell types and endothelial cells), the distribution of heterochromatin grains, and the staining of euchromatin in the nucleus. Rounded and darkly stained nuclei with 2–4 granules of heterochromatin, often with a perinuclear halo and/or a pinkish crescent of cytoplasm, are typical of oligodendrocytes; in the human, some oligodendrocytes have clear nuclei with lightly stained euchromatin. Elongated, comma-shaped or polylobular darkly stained nuclei with numerous small granules forming a grid across the nucleus, often with greenish inclusions next to the nucleus (in the unstained cytoplasm), are typical of microglia. Cells with lightly stained nuclei and unstained cytoplasm, with a rim of peripheral heterochromatin under the nuclear envelope and several heterochromatin granules attached to this rim or in the heterochromatin net, were classified as astrocytes. Some astrocytes have yellow inclusions and pinkish threads in the perinuclear cytoplasm. Homogeneous staining of the euchromatin helps make the distinction between astrocytes and endothelial cells. Lightly stained nuclei and stained cytoplasm are the features of neurons. Nuclei with an “empty” appearance and small granules of heterochromatin around a distinct nucleolus are typical of large pyramids as well as of large nonpyramidal neurons, like fusiform von Economo neurons. Lightly stained nuclei with a nucleolus partially or totally surrounded by irregular clumps of heterochromatin and 1–2 additional heterochromatin granules in the nucleoplasm are typical of small neurons. Nuclear folding, present in neurons, additionally helped distinguish small neurons from astrocytes, especially in humans.

The counting frame (disector) size for cell counts was 50–60 μm. To ensure an unbiased estimate of the number of cells, we first measured the thickness of each section and set a guard zone at the bottom and top of each section to correct for objects plucked during sectioning (minimum 2 μm in 10–15-μm sections after tissue shrinkage); the disector thickness was thus smaller than the thickness of the section [166–168]. The height of the counting frame was 5 μm, and grid spacing was 100–300 μm. Cells were counted if their nuclei fell within the counting frame or touched the 2 inclusion lines, but not the 2 exclusion lines [168]. These parameters yielded a sampling fraction with a coefficient of error of <10% per contour, as recommended [167, 168]. The use of uniform random sampling ensured that every part of each area examined had the same chance of being included in the sample. We computed cell density by dividing the estimated number of counted cells with the estimated volume of each contour to assess the relative density (cells/volume of all layers in mm^3^) and the packing density (cells/volume of each layer in mm^3^) in each area and each human subject and rhesus monkey. The relative laminar density of neurons is an indicator of the average density of neurons within a cortical gray matter column and shows which layers have more neurons. On the other hand, the packing laminar density of neurons, which was estimated as the average density of neurons in each layer divided by the volume (mm^3^) of that layer, shows which layers have more densely packed populations of neurons within a cortical gray matter column of set volume.

We estimated the density of axons and the thickness of axons and myelin in the white matter at the EM in humans and rhesus monkeys. We sampled a volume of approximately 1 cm^3^ below each prefrontal cortical area, using a systematic random sampling fraction of 1:1,000 that yielded more than 2,000 axons, per human subject and rhesus monkey, per area. We divided the white matter (as described above) into a superficial part (close to the gray matter) and a deep part. We captured high-resolution images of areas of interest that were imported in ImageJ and calibrated. We estimated the overall density of axons at low magnification (2,000×–3,300×) by dividing the surface area of axon profiles by the total surface area of the sampled region. We estimated the inner and outer diameter as well as the thickness of the surrounding myelin sheath at high magnification (10,000×), as described [64]. In our analysis, we included all axon profiles: those that were perpendicular to the cutting plane and appeared cylindrical, as well as elongated profiles. To ensure consistency, we measured the diameter perpendicular to the center of the maximum diameter of the axon profile.

### Optical density analysis of intracortical myelin

We quantified the laminar myelin content from images of representative columns from each area in the human and monkey PFC captured at the light microscope (Olympus BX 51), under brightfield, with a CCD camera (Olympus DP70), connected to a workstation running imaging software (DP Controller, Olympus). We captured images using a UPlanFl 10× /0.30 lens with the same light exposure to minimize background variability. We obtained optical density measurements from 5–15 images of cortical gray matter columns taken from at least 3 sections per area in each human subject or rhesus monkey. We imported images into ImageJ, converted them into gray scale, and inverted them. We measured levels of background staining in gray matter regions with no myelinated axons within each image and subtracted background pixel values from each image to eliminate staining inconsistencies, due to experimental variability. These gray matter regions with few or no myelinated axons were typically in the superficial, supragranular layers of the cortex (layers 1–3) in each image of a cortical column. As a result, pixels with strong staining had consistently higher optical density values than the background. We measured the mean gray level density for each normalized image and obtained overall mean gray level values for each area. We also estimated the gray level density along the depth of background-corrected columns, encompassing cortical thickness from the pial surface to the white matter. To normalize gray level density profiles to a standard depth for all cortical regions, we divided each profile into 20 bins and averaged density values across images for each bin.

### Connection dataset

We analyzed an extensive connection dataset that includes quantitative information on the existence or absence and numbers of labeled projection neurons in the cortex of macaque monkeys after injections of retrograde neural tracers in the ACC, OFC, and LPFC. These data were recompiled from databases used in published studies from our laboratory [e.g., [107, 108, 157]; see S2 Table for a detailed list of publications with these data].

### Relative projection frequencies according to spatial adjacency and structural type similarity

Labeled projection neurons were plotted throughout the entire cortex from a series of sections from each animal (separated by 400–1,000 μm), using a commercially available semiautomated system (Neurolucida-Stereoinvestigator; Microbrightfield). For earlier studies, we used a custom-built system that included an analog/digital-microscope/computer interface (Nikon, Optiphot/Austin/i486 PC running DOS) with plotting software developed in our laboratory [156]. We estimated relative frequencies of connections that were present in each animal by dividing the number of labeled projection neurons in an area by the total number of labeled projection neurons across the entire cortex. Analysis was limited to ipsilateral connections. This procedure normalized connectivity data and minimized variability due to experimental differences across rhesus monkeys (e.g., size of tracer injection or tracer transport).

For the analysis of short- and long-range ipsilateral corticocortical connections of ACC, OFC, and LPFC, we classified ratios of projection neurons based on their spatial adjacency (distance; S3 Table). Connections with areas in the frontal lobe, including prefrontal and premotor areas, were considered short-range connections. All other connections with projection neurons in the temporal, parietal, and occipital lobes were considered to be long-range connections. This classification of connections is largely in line with previous studies that have described or analyzed these and other connections and datasets in primates and other species [4, 28, 37, 72, 73, 118].

We also classified cortical areas that innervated ACC, OFC, and LPFC, based on their structural type (S3 Table). This classification is based first on observations that cortical areas tend to connect mainly with other cortical areas of similar structure [74, 107, 169], and second, it is based on the Structural Model of Connections, which states that the laminar structural similarity of cortical areas is related to the laminar origin and termination pattern of connections between them, as well as their relative strength (e.g., [21, 73, 107, 108, 113, 114, 118]). Classical and recent studies have determined the structural (dis)similarity of areas based on qualitative terms or quantitative measurements of several features, including the number and definition of individual cortical layers, assessed in Nissl- or myelin-stained sections, neuronal density, or the density of various receptors [21, 23, 27, 34, 38, 44, 45, 170]. Laminar definition and the absence or the presence, as well as the thickness, of layer 4 have been useful for distinguishing cytoarchitectonic features. Neurons in cortical layer 4 are small, granular, and densely packed and can be further distinguished by cellular and neurochemical criteria, like the lack of labeling with an antibody for a neurofilament protein (SMI-32), which labels neurons in layers 3 and 5 [42, 43, 162]. Pyramidal neurons in adjacent layers 3 and 5 are also considerably larger and not as densely packed.

Using these criteria, we defined 3 major cortical types that have been consistently and widely used in the literature and assigned cortical areas in frontal, temporal, parietal, and occipital lobes in one of these categories (S3 Table). The first structural type of cortex includes agranular areas, identified as those that lack layer 4 altogether. The second type includes dysgranular areas, which have a poorly developed layer 4. Finally, the third type includes eulaminate cortical areas, which describes the rest of the areas with 6 well-defined layers and laminar elaboration. Areas classified based on these 3 structural types also mostly differ quantitatively in the laminar density of neurons. The density of neurons in association cortices gradually increases from agranular to dysgranular to eulaminate areas with small variations, and agranular and dysgranular (limbic) areas have a lower density of neurons in upper layers 2 and 3 compared to deep layers 5 and 6.

### Statistical analysis

We gathered data blind to condition and cortical region in the part of the study involving human postmortem tissue. Random codes for human subjects and images were broken after completion of each part of the study. In most instances, data collection was performed by at least 2 investigators. We employed 1-way ANOVA for overall comparison of cell or optical densities and estimates across species, areas, and laminar groups. For analyses that showed significant differences (*p* < 0.05), we performed post hoc pair comparisons (Bonferroni method). Data were tabulated in Excel (Office 365, Microsoft), and analyses were performed using Statistica (StatSoft, Tulsa, Oklahoma, US; RRID: SCR_014213).

For the EM analysis of axon densities and sizes, we obtained samples from widely spaced ultrathin sections (1 every 10) and fields of view through systematic random sampling to minimize the likelihood of sampling axons from the same parent branch. This sampling scheme and the fact that most axons branch very close to or after they enter the gray matter minimized the likelihood of counting segments of the same axon more than once. We evaluated data through scatter and frequency distribution plots and K-means cluster analysis with parameters set to maximize initial between-cluster distances. Data distributions for continuous variables were not significantly different from normal as determined by the Kolmogorov–Smirnov test and thus allowed the use of parametric statistics. We initially used x^2^ and Kolmogorov–Smirnov tests to examine axon size distributions and multiple linear regression analysis to examine correlations.

To demonstrate global similarities/differences among prefrontal areas, we performed NMDS, which allows visualization of high-dimensional data into a low 2-dimensional space that approximates pairwise distances between data points. We used all estimated variables at the light and electron microscopic level. These included 34 architectural features, like the relative and packing laminar density of neurons, oligodendrocytes, and intracortical myelin, as well as 12 ultrastructural features of the white matter, including relative axon density (all myelinated axons), outer diameter, myelin thickness, g-ratio, and relative proportion of thin, medium, thick, and extra-large axons. The relative proximity among items in an NMDS diagram represents their relative similarity. For each of the 3 brain regions in the 2 species, the values were averaged to produce a feature vector comprised of 34 features derived from brightfield analysis (neuron and oligodendroglia density all layers, relative and packing laminar density of neurons and oligodendroglia, and relative laminar density of myelin). This feature set was z-scored and used to create a distance matrix, which served as the basis for nonmetric multidimensional scaling. This technique allows high-dimensional data to be assigned to new lower-dimensional coordinates that preserve distances between data points. We thus projected the 34-dimensional feature data into a 2-dimensional space. We performed a similar analysis for the EM measurements in 6 white matter regions (SWM and DWM below ACC, OFC, and LPFC) and 12 feature dimensions (axon density, outer diameter, myelin thickness, g-ratio, ratio of thin axons, ratio of medium axons, ratio of thick axons, ratio of XLarge axons, myelin thickness of thin axons, myelin thickness of medium axons, myelin thickness of thick axons, and myelin thickness of XLarge axons). We additionally performed discriminant analysis to identify experimental measures that minimize the overlap and clearly separate the distributions of individual data points belonging to different cortical areas across species. Moreover, we performed hierarchical cluster analysis (HCA) to group areas based on (dis)similarities in their parameter profiles. In this test, the relative similarity of areas is expressed as the distance between two branching points in a cluster tree diagram. HCA and NMDS analyses employed squared area (dis)similarity matrices derived from the normalized area profiles by Pearson′s correlation. In addition, we examined whether the feature set of each region between species was correlated. As the feature set included values that vary considerably in scale, we log-transformed the data and then regressed the monkey feature set onto the human feature set.

Finally, we examined the potential effects of sex, PMI, age at death, and other diagnoses (i.e., seizures) on all estimates for axon size as well as axon and cell density, using correlation analysis. In addition, we compared all estimated variables between and within species using MANCOVA with sex, PMI, age at death, and other diagnoses as covariates. These analyses did not yield significant effects.

### Photography

Captured digital images that were used for analyses were not modified. Images used in the figures were imported into Adobe Illustrator CC software (Adobe Systems, San José, California, US) to assemble in panels. Minor adjustments of overall brightness and contrast were made, but the images were not retouched.

## Supporting information

S1 Data Data used for figures. (XLSX)

S1 Table Autism diagnostic interview—Revised (ADI-R) scores for autistic subjects (from https://atpportal.org). (DOCX)

S2 Table Data on rhesus monkeys, tracers, and injection sites used for cortical connectivity analysis and a list of quantitative tract-tracing studies that presented this data. (DOCX)

S3 Table Cortical areas grouped by lobe and structural type that are connected with anterior cingulate cortex (ACC), orbitofrontal cortex (OFC), and lateral prefrontal cortex (LPFC), based on tract-tracing studies in rhesus macaques. (DOCX)

## Abbreviations

ACC: anterior cingulate cortex
ADI-R: Autism Diagnostic Interview–Revised
ASD: autism spectrum disorder
DWM: deep white matter
EM: electron microscope
LPFC: lateral prefrontal cortex
MANCOVA: multivariate analysis of covariance
NMDS: nonmetric multidimensional scaling
OFC: orbitofrontal cortex
OPro: orbital proisocortex
PFC: prefrontal cortex
PMI: postmortem interval
SWM: superficial white matter

## References

[1] Schenker NM, Desgouttes AM, Semendeferi K. Neural connectivity and cortical substrates of cognition in hominoids. J HumEvol. 2005;49(5):547–69. 10.1016/j.jhevol.2005.06.004 16076478

[2] Park HJ, Friston K. Structural and functional brain networks: from connections to cognition. Science. 2013;342(6158):1238411 doi: 10.1126/science.1238411 24179229 10.1126/science.1238411

[3] Huntenburg JM, Bazin PL, Goulas A, Tardif CL, Villringer A, Margulies DS. A Systematic Relationship Between Functional Connectivity and Intracortical Myelin in the Human Cerebral Cortex. Cereb Cortex. 2017;27(2):981–97. doi: 10.1093/cercor/bhx030 28184415 10.1093/cercor/bhx030 PMC5390400

[4] Horvat S, Gamanut R, Ercsey-Ravasz M, Magrou L, Gamanut B, Van Essen DC, et al Spatial Embedding and Wiring Cost Constrain the Functional Layout of the Cortical Network of Rodents and Primates. PLoS Biol. 2016;14(7):e1002512 doi: 10.1371/journal.pbio.1002512 27441598 10.1371/journal.pbio.1002512 PMC4956175

[5] Glasser MF, Coalson TS, Robinson EC, Hacker CD, Harwell J, Yacoub E, et al A multi-modal parcellation of human cerebral cortex. Nature. 2016;536(7615):171–8. doi: 10.1038/nature18933 27437579 10.1038/nature18933 PMC4990127

[6] Sporns O, Tononi G, Kotter R. The human connectome: A structural description of the human brain. PLoS Comput Biol. 2005;1(4):e42 Epub 2005/10/05. doi: 10.1371/journal.pcbi.0010042 16201007 10.1371/journal.pcbi.0010042 PMC1239902

[7] Croxson PL, Johansen-Berg H, Behrens TE, Robson MD, Pinsk MA, Gross CG, et al Quantitative investigation of connections of the prefrontal cortex in the human and macaque using probabilistic diffusion tractography. J Neurosci. 2005;25(39):8854–66. doi: 10.1523/JNEUROSCI.1311-05.2005 16192375 10.1523/JNEUROSCI.1311-05.2005 PMC6725599

[8] Yeo BT, Krienen FM, Sepulcre J, Sabuncu MR, Lashkari D, Hollinshead M, et al The organization of the human cerebral cortex estimated by intrinsic functional connectivity. J Neurophysiol. 2011;106(3):1125–65. doi: 10.1152/jn.00338.2011 21653723 10.1152/jn.00338.2011 PMC3174820

[9] Thomas C, Ye FQ, Irfanoglu MO, Modi P, Saleem KS, Leopold DA, et al Anatomical accuracy of brain connections derived from diffusion MRI tractography is inherently limited. Proc Natl Acad Sci USA. 2014;111(46):16574–9. doi: 10.1073/pnas.1405672111 25368179 10.1073/pnas.1405672111 PMC4246325

[10] Jbabdi S, Sotiropoulos SN, Haber SN, Van Essen DC, Behrens TE. Measuring macroscopic brain connections in vivo. Nat Neurosci. 2015;18(11):1546–55. doi: 10.1038/nn.4134 26505566 10.1038/nn.4134

[11] Reveley C, Seth AK, Pierpaoli C, Silva AC, Yu D, Saunders RC, et al Superficial white matter fiber systems impede detection of long-range cortical connections in diffusion MR tractography. Proc Natl Acad Sci USA. 2015;112(21):E2820–8. doi: 10.1073/pnas.1418198112 25964365 10.1073/pnas.1418198112 PMC4450402

[12] Mortazavi F, Oblak AL, Morrison WZ, Schmahmann JD, Stanley HE, Wedeen VJ, et al Geometric Navigation of Axons in a Cerebral Pathway: Comparing dMRI with Tract Tracing and Immunohistochemistry. Cereb Cortex. 2017:1–14. 10.1093/cercor/bhx034 PMC6074943 28203748

[13] Zikopoulos B, Hoistad M, John Y, Barbas H. Posterior orbitofrontal and anterior cingulate pathways to the amygdala target inhibitory and excitatory systems with opposite functions. J Neurosci. 2017;37(20):14. 10.1523/JNEUROSCI.3940-16.2017 PMC5444191 28411274

[14] Bunce JG, Zikopoulos B, Feinberg M, Barbas H. Parallel prefrontal pathways reach distinct excitatory and inhibitory systems in memory-related rhinal cortices. J Comp Neurol. 2013;512(18):4260–83. 10.1002/cne.23413 PMC3881238 23839697

[15] Xiao D, Zikopoulos B, Barbas H. Laminar and modular organization of prefrontal projections to multiple thalamic nuclei. Neuroscience. 2009;161(4):1067–81. doi: 10.1016/j.neuroscience.2009.04.034 19376204 10.1016/j.neuroscience.2009.04.034 PMC2700123

[16] Zikopoulos B, Barbas H. Pathways for emotions and attention converge on the thalamic reticular nucleus in primates. J Neurosci. 2012;32(15):5338–50. Epub 2012/04/13. doi: 10.1523/JNEUROSCI.4793-11.2012 22496579 10.1523/JNEUROSCI.4793-11.2012 PMC3342673

[17] Jbabdi S, Lehman JF, Haber SN, Behrens TE. Human and monkey ventral prefrontal fibers use the same organizational principles to reach their targets: tracing versus tractography. J Neurosci. 2013;33(7):3190–201. doi: 10.1523/JNEUROSCI.2457-12.2013 23407972 10.1523/JNEUROSCI.2457-12.2013 PMC3602794

[18] Lehman JF, Greenberg BD, McIntyre CC, Rasmussen SA, Haber SN. Rules Ventral Prefrontal Cortical Axons Use to Reach Their Targets: Implications for Diffusion Tensor Imaging Tractography and Deep Brain Stimulation for Psychiatric Illness. J Neurosci. 2011;31(28):10392–402. doi: 10.1523/JNEUROSCI.0595-11.2011 21753016 10.1523/JNEUROSCI.0595-11.2011 PMC3445013

[19] Schmahmann JD, Pandya DN, Wang R, Dai G, D′Arceuil HE, de Crespigny AJ, et al Association fibre pathways of the brain: parallel observations from diffusion spectrum imaging and autoradiography. Brain. 2007;130(Pt 3):630–53. doi: 10.1093/brain/awl359 17293361 10.1093/brain/awl359

[20] Maier-Hein K, Neher P, Houde J-C, Cote M-A, Garyfallidis E, Zhong J, et al Tractography-based connectomes are dominated by false-positive connections. bioRxiv. 2016.

[21] Barbas H. General Cortical and special Prefrontal Connections: Principles from Structure to Function. Annu Rev Neurosci. 2015;38:269–89. doi: 10.1146/annurev-neuro-071714-033936 25897871 10.1146/annurev-neuro-071714-033936

[22] Barbas H, García-Cabezas MA. How the prefrontal executive got its stripes. Curr Opin Neurobiol. 2016;40:125–34. doi: 10.1016/j.conb.2016.07.003 27479655 10.1016/j.conb.2016.07.003 PMC5056826

[23] Schleicher A, Palomero-Gallagher N, Morosan P, Eickhoff SB, Kowalski T, de Vos K, et al Quantitative architectural analysis: a new approach to cortical mapping. Anat Embryol (Berl). 2005;210(5–6):373–86. 16249867 10.1007/s00429-005-0028-2

[24] Burt JB, Demirtas M, Eckner WJ, Navejar NM, Ji JL, Martin WJ, et al Hierarchy of transcriptomic specialization across human cortex captured by myelin map topography. bioRxiv. 2017. 10.1038/s41593-018-0195-0 PMC6119093 30082915

[25] Amunts K, Lepage C, Borgeat L, Mohlberg H, Dickscheid T, Rousseau ME, et al BigBrain: an ultrahigh-resolution 3D human brain model. Science. 2013;340(6139):1472–5. Epub 2013/06/22. doi: 10.1126/science.1235381 23788795 10.1126/science.1235381

[26] Goulas A, Werner R, Beul SF, Saering D, van den Heuvel M, Triarhou LC, et al Cytoarchitectonic similarity is a wiring principle of the human connectome. bioRxiv. 2016.

[27] Amunts K, Zilles K. Architectonic Mapping of the Human Brain beyond Brodmann. Neuron. 2015;88(6):1086–107. doi: 10.1016/j.neuron.2015.12.001 26687219 10.1016/j.neuron.2015.12.001

[28] Schmahmann JD, Pandya DN. Fiber pathways of the brain. New York: Oxford University Press; 2006.

[29] Petrides M, Pandya DN. Dorsolateral prefrontal cortex: comparative cytoarchitectonic analysis in the human and the macaque brain and corticocortical connection patterns. Eur J Neurosci. 1999;11:1011–36. 10103094 10.1046/j.1460-9568.1999.00518.x

[30] Petrides M, Pandya DN. Comparative cytoarchitectonic analysis of the human and the macaque ventrolateral prefrontal cortex and corticocortical connection patterns in the monkey. Eur J Neurosci. 2002;16(2):291–310. 12169111 10.1046/j.1460-9568.2001.02090.x

[31] Thiebaut de Schotten M, Dell′Acqua F, Valabregue R, Catani M. Monkey to human comparative anatomy of the frontal lobe association tracts. Cortex. 2012;48(1):82–96. doi: 10.1016/j.cortex.2011.10.001 22088488 10.1016/j.cortex.2011.10.001

[32] Zhang K, Sejnowski TJ. A universal scaling law between gray matter and white matter of cerebral cortex. Proc Natl Acad Sci USA. 2000;97(10):5621–6. doi: 10.1073/pnas.090504197 10792049 10.1073/pnas.090504197 PMC25878

[33] Oishi K, Huang H, Yoshioka T, Ying SH, Zee DS, Zilles K, et al Superficially located white matter structures commonly seen in the human and the macaque brain with diffusion tensor imaging. Brain connectivity. 2011;1(1):37–47. doi: 10.1089/brain.2011.0005 22432953 10.1089/brain.2011.0005 PMC3569096

[34] Dombrowski SM, Hilgetag CC, Barbas H. Quantitative architecture distinguishes prefrontal cortical systems in the rhesus monkey. Cereb Cortex. 2001;11:975–88. 11549620 10.1093/cercor/11.10.975

[35] von Economo C. Cellular structure of the human cerebral cortex (Translated and edited by Thriarhou Lazaros C.). Basel (Switzerland): Karger; 1927/2009.

[36] Barbas H, Zikopoulos B, Timbie C. Sensory pathways and emotional context for action in primate prefrontal cortex. Biol Psychiatry. 2011;69(12):1133–9. doi: 10.1016/j.biopsych.2010.08.008 20889144 10.1016/j.biopsych.2010.08.008

[37] Yeterian EH, Pandya DN, Tomaiuolo F, Petrides M. The cortical connectivity of the prefrontal cortex in the monkey brain. Cortex. 2012;48(1):58–81. doi: 10.1016/j.cortex.2011.03.004 21481342 10.1016/j.cortex.2011.03.004 PMC3161133

[38] Barbas H, Pandya DN. Architecture and intrinsic connections of the prefrontal cortex in the rhesus monkey. J Comp Neurol. 1989;286(3):353–75. doi: 10.1002/cne.902860306 2768563 10.1002/cne.902860306

[39] Cavada C, Company T, Tejedor J, Cruz-Rizzolo RJ, Reinoso-Suarez F. The anatomical connections of the macaque monkey orbitofrontal cortex. A review. Cereb Cortex. 2000;10:220–42. 10731218 10.1093/cercor/10.3.220

[40] Goldman-Rakic PS. Circuitry of primate prefrontal cortex and regulation of behavior by representational memory In: Mountcastle V, Plum F, editors. Handbook of Physiology—The Nervous System, Higher Functions of the Brain, Vol 5 Washington D.C.: American Physiological Society; 1987 p. 373–417.

[41] Fuster JM. The prefrontal cortex. 4th ed London (UK): Elsevier/Academic Press; 2008.

[42] García-Cabezas MA, Barbas H. Area 4 has layer IV in adult primates. Eur J Neurosci. 2014;39:1824–34. doi: 10.1111/ejn.12585 24735460 10.1111/ejn.12585 PMC4201116

[43] Barbas H, García-Cabezas MA. Motor cortex layer 4: less is more. Trends Neurosci. 2015;38(5):259–61. doi: 10.1016/j.tins.2015.03.005 25868984 10.1016/j.tins.2015.03.005 PMC4500633

[44] von Economo C, Koskinas GN. Atlas of cytoarchitectonics of the adult human cerebral cortex. Translated from the German original, revised and edited with an Introduction and additional appendix material by Triarhou L. C.. 1st English ed Basel; New York: Karger; 1925/2008 x, 182 p. p.

[45] Sanides F. Functional architecture of motor and sensory cortices in primates in the light of a new concept of neocortex evolution In: Noback CR, Montagna W, editors. The Primate Brain: Advances in Primatology. New York (NY): Appleton-Century-Crofts Educational Division/Meredith Corporation; 1970 p. 137–208.

[46] Palomero-Gallagher N, Eickhoff SB, Hoffstaedter F, Schleicher A, Mohlberg H, Vogt BA, et al Functional organization of human subgenual cortical areas: Relationship between architectonical segregation and connectional heterogeneity. Neuroimage. 2015;115:177–90. doi: 10.1016/j.neuroimage.2015.04.053 25937490 10.1016/j.neuroimage.2015.04.053 PMC4801475

[47] Palomero-Gallagher N, Vogt BA, Schleicher A, Mayberg HS, Zilles K. Receptor architecture of human cingulate cortex: evaluation of the four-region neurobiological model. Hum Brain Mapp. 2009;30(8):2336–55. Epub 2008/11/27. doi: 10.1002/hbm.20667 19034899 10.1002/hbm.20667 PMC6870973

[48] Vogt BA, Hof PR, Zilles K, Vogt LJ, Herold C, Palomero-Gallagher N. Cingulate area 32 homologies in mouse, rat, macaque and human: cytoarchitecture and receptor architecture. J Comp Neurol. 2013;521(18):4189–204. doi: 10.1002/cne.23409 23840027 10.1002/cne.23409

[49] Palomero-Gallagher N, Zilles K, Schleicher A, Vogt BA. Cyto- and receptor architecture of area 32 in human and macaque brains. J Comp Neurol. 2013;521(14):3272–86. doi: 10.1002/cne.23346 23787873 10.1002/cne.23346

[50] Joyce MP, Barbas H. Cortical connections position primate area 25 as a keystone for interoception, emotion, and memory. J Neurosci. Forthcoming 2018 doi: 10.1523/JNEUROSCI.2363-17.2017 29358365 10.1523/JNEUROSCI.2363-17.2017 PMC5815452

[51] Petrides M, Tomaiuolo F, Yeterian EH, Pandya DN. The prefrontal cortex: comparative architectonic organization in the human and the macaque monkey brains. Cortex. 2012;48(1):46–57. doi: 10.1016/j.cortex.2011.07.002 21872854 10.1016/j.cortex.2011.07.002

[52] Mackey S, Petrides M. Architecture and morphology of the human ventromedial prefrontal cortex. Eur J Neurosci. 2014;40(5):2777–96. doi: 10.1111/ejn.12654 25123211 10.1111/ejn.12654

[53] Barbas H, Zikopoulos B. Sequential and parallel circuits for emotional processing in primate orbitofrontal cortex In: David Z, Scott R, editors. The Orbitofrontal Cortex. Oxford: Oxford University Press; 2006 p. 57–91.

[54] Mackey S, Petrides M. Architectonic mapping of the medial region of the human orbitofrontal cortex by density profiles. Neuroscience. 2009;159(3):1089–107. doi: 10.1016/j.neuroscience.2009.01.036 19356690 10.1016/j.neuroscience.2009.01.036

[55] Uylings HB, Sanz-Arigita EJ, de Vos K, Pool CW, Evers P, Rajkowska G. 3-D cytoarchitectonic parcellation of human orbitofrontal cortex correlation with postmortem MRI. Psychiatry Res. 2010;183(1):1–20. doi: 10.1016/j.pscychresns.2010.04.012 20538437 10.1016/j.pscychresns.2010.04.012 PMC2902628

[56] García-Cabezas MA, Barbas H. Anterior Cingulate Pathways May Affect Emotions Through Orbitofrontal Cortex. Cereb Cortex. 2017;27(10):4891–910. doi: 10.1093/cercor/bhw284 27655930 10.1093/cercor/bhw284 PMC6075591

[57] Medalla M, Barbas H. Diversity of laminar connections linking periarcuate and lateral intraparietal areas depends on cortical structure. Eur J Neurosci. 2006;23(1):161–79. doi: 10.1111/j.1460-9568.2005.04522.x 16420426 10.1111/j.1460-9568.2005.04522.x

[58] Petrides M. Lateral prefrontal cortex: architectonic and functional organization. PhilosTransRSocLond B BiolSci. 2005;360(1456):781–95. 10.1098/rstb.2005.1631 PMC1569489 15937012

[59] Rajkowska G, Goldman-Rakic PS. Cytoarchitectonic definition of prefrontal areas in the normal human cortex:I. Remapping of areas 9 and 46 using quantitative criteria. Cereb Cortex. 1995;5:307–22. 7580124 10.1093/cercor/5.4.307

[60] Glasser MF, Van Essen DC. Mapping Human Cortical Areas In Vivo Based on Myelin Content as Revealed by T1- and T2-Weighted MRI. J Neurosci. 2011;31(32):11597–616. doi: 10.1523/JNEUROSCI.2180-11.2011 21832190 10.1523/JNEUROSCI.2180-11.2011 PMC3167149

[61] Wang SS, Shultz JR, Burish MJ, Harrison KH, Hof PR, Towns LC, et al Functional trade-offs in white matter axonal scaling. J Neurosci. 2008;28(15):4047–56. doi: 10.1523/JNEUROSCI.5559-05.2008 18400904 10.1523/JNEUROSCI.5559-05.2008 PMC2779774

[62] Kimura F, Itami C. Myelination and isochronicity in neural networks. Front Neuroanat. 2009;3:12 doi: 10.3389/neuro.05.012.2009 19597561 10.3389/neuro.05.012.2009 PMC2708965

[63] Zikopoulos B, Barbas H. Altered neural connectivity in excitatory and inhibitory cortical circuits in autism. Front Hum Neurosci. 2013;7:609 Epub 2013/10/08. doi: 10.3389/fnhum.2013.00609 24098278 10.3389/fnhum.2013.00609 PMC3784686

[64] Zikopoulos B, Barbas H. Changes in prefrontal axons may disrupt the network in autism. J Neurosci. 2010;30(44):14595–609. doi: 10.1523/JNEUROSCI.2257-10.2010 21048117 10.1523/JNEUROSCI.2257-10.2010 PMC3073590

[65] Liewald D, Miller R, Logothetis N, Wagner HJ, Schuz A. Distribution of axon diameters in cortical white matter: an electron-microscopic study on three human brains and a macaque. Biol Cybern. 2014;108(5):541–57. doi: 10.1007/s00422-014-0626-2 25142940 10.1007/s00422-014-0626-2 PMC4228120

[66] LaMantia AS, Rakic P. Cytological and quantitative characteristics of four cerebral commissures in the rhesus monkey. J Comp Neurol. 1990;291:520–37. doi: 10.1002/cne.902910404 2329189 10.1002/cne.902910404

[67] Bullmore E, Sporns O. The economy of brain network organization. Nat Rev Neurosci. 2012;13(5):336–49. Epub 2012/04/14. doi: 10.1038/nrn3214 22498897 10.1038/nrn3214

[68] Ramón y Cajal S. Histology of the nervous system of man and vertebrates, volume I. Translated from French by Neely Swanson and Larry W. Swanson. Swanson N, Swanson LW, editors. New York: Oxford University Press; 1909/1995.

[69] Rushton WAH. A theory of the effects of fibre size in medullated nerve. Journal of Physiology. 1951;115(1):101–22. 14889433 10.1113/jphysiol.1951.sp004655 PMC1392008

[70] Jones EG, Coulter JD, Hendry SHC. Intracortical connectivity of architectonic fields in the somatic sensory, motor and parietal cortex of monkeys. J Comp Neurol. 1978;181:291–348. doi: 10.1002/cne.901810206 99458 10.1002/cne.901810206

[71] Hilgetag CC, Barbas H. Role of mechanical factors in the morphology of the primate cerebral cortex. PLoS Comput Biol. 2006;2(3):e22 doi: 10.1371/journal.pcbi.0020022 16557292 10.1371/journal.pcbi.0020022 PMC1409812

[72] Ercsey-Ravasz M, Markov NT, Lamy C, Van Essen DC, Knoblauch K, Toroczkai Z, et al A predictive network model of cerebral cortical connectivity based on a distance rule. Neuron. 2013;80(1):184–97. doi: 10.1016/j.neuron.2013.07.036 24094111 10.1016/j.neuron.2013.07.036 PMC3954498

[73] Hilgetag CC, Medalla M, Beul S, Barbas H. The primate connectome in context: principles of connections of the cortical visual system. NeuroImage. 2016;134:685–702. doi: 10.1016/j.neuroimage.2016.04.017 27083526 10.1016/j.neuroimage.2016.04.017 PMC5135480

[74] Petrides M, Pandya DN. Association fiber pathways to the frontal cortex from the superior temporal region in the rhesus monkey. J Comp Neurol. 1988;273:52–66. doi: 10.1002/cne.902730106 2463275 10.1002/cne.902730106

[75] Rempel-Clower NL, Barbas H. The laminar pattern of connections between prefrontal and anterior temporal cortices in the rhesus monkey is related to cortical structure and function. Cereb Cortex. 2000;10(9):851–65. 10982746 10.1093/cercor/10.9.851

[76] Reep R. Relationship between prefrontal and limbic cortex: A comparative and anatomical review. Brain Behavior and Evolution. 1984;25:1–80. 10.1159/000118849 6398115

[77] Pandya D, Seltzer B, Petrides M, Cipolloni PB. Cerebral Cortex: Architecture, Connections, and the Dual Origin Concept. New York: Oxford University Press; 2015.

[78] Peters A. The effects of normal aging on myelinated nerve fibers in monkey central nervous system. Front Neuroanat. 2009;3:11 doi: 10.3389/neuro.05.011.2009 19636385 10.3389/neuro.05.011.2009 PMC2713738

[79] Peters A. The effects of normal aging on myelin and nerve fibers: a review. Journal of Neurocytology. 2002;31(8–9):581–93. 14501200 10.1023/a:1025731309829

[80] Liu H, Yang Y, Xia Y, Zhu W, Leak RK, Wei Z, et al Aging of cerebral white matter. Ageing research reviews. 2017;34:64–76. doi: 10.1016/j.arr.2016.11.006 27865980 10.1016/j.arr.2016.11.006 PMC5250573

[81] Bennett IJ, Madden DJ. Disconnected aging: cerebral white matter integrity and age-related differences in cognition. Neuroscience. 2014;276:187–205. doi: 10.1016/j.neuroscience.2013.11.026 24280637 10.1016/j.neuroscience.2013.11.026 PMC4032380

[82] Lamar M, Zhou XJ, Charlton RA, Dean D, Little D, Deoni SC. In vivo quantification of white matter microstructure for use in aging: a focus on two emerging techniques. The American journal of geriatric psychiatry: official journal of the American Association for Geriatric Psychiatry. 2014;22(2):111–21. 24080382 10.1016/j.jagp.2013.08.001 PMC3947219

[83] Gunning-Dixon FM, Brickman AM, Cheng JC, Alexopoulos GS. Aging of cerebral white matter: a review of MRI findings. International journal of geriatric psychiatry. 2009;24(2):109–17. doi: 10.1002/gps.2087 18637641 10.1002/gps.2087 PMC2631089

[84] Goulas A, Bastiani M, Bezgin G, Uylings HB, Roebroeck A, Stiers P. Comparative analysis of the macroscale structural connectivity in the macaque and human brain. PLoS Comput Biol. 2014;10(3):e1003529 doi: 10.1371/journal.pcbi.1003529 24676052 10.1371/journal.pcbi.1003529 PMC3967942

[85] Semendeferi K, Damasio H, Frank R, Van Hoesen GW. The evolution of the frontal lobes: a volumetric analysis based on three-dimensional reconstructions of magnetic resonance scans of human and ape brains. Journal of Human Evolution. 1997;32:375–88. doi: 10.1006/jhev.1996.0099 9085187 10.1006/jhev.1996.0099

[86] Cole MW, Yeung N, Freiwald WA, Botvinick M. Cingulate cortex: Diverging data from humans and monkeys. Trends in Neurosciences. 2009;32(11):566–74. doi: 10.1016/j.tins.2009.07.001 19781794 10.1016/j.tins.2009.07.001 PMC7580873

[87] Sereno MI, Tootell RBH. From monkeys to humans: what do we now know about brain homologies? Current Opinion in Neurobiology. 2005;15(2):135–44. doi: 10.1016/j.conb.2005.03.014 15831394 10.1016/j.conb.2005.03.014

[88] Passingham R. How good is the macaque monkey model of the human brain? Current Opinion in Neurobiology. 2009;19(1):6–11. doi: 10.1016/j.conb.2009.01.002 19261463 10.1016/j.conb.2009.01.002 PMC2706975

[89] Yopak KE, Lisney TJ, Darlington RB, Collin SP, Montgomery JC, Finlay BL. A conserved pattern of brain scaling from sharks to primates. Proc Natl Acad Sci USA. 2010;107(29):12946–51. Epub 2010/07/10. doi: 10.1073/pnas.1002195107 20616012 10.1073/pnas.1002195107 PMC2919912

[90] Sherwood CC, Stimpson CD, Raghanti MA, Wildman DE, Uddin M, Grossman LI, et al Evolution of increased glia-neuron ratios in the human frontal cortex. Proc Natl Acad Sci USA. 2006;103(37):13606–11. doi: 10.1073/pnas.0605843103 16938869 10.1073/pnas.0605843103 PMC1564260

[91] Herculano-Houzel S. The human brain in numbers: a linearly scaled-up primate brain. Front Hum Neurosci. 2009;3:31 doi: 10.3389/neuro.09.031.2009 19915731 10.3389/neuro.09.031.2009 PMC2776484

[92] Azevedo FA, Carvalho LR, Grinberg LT, Farfel JM, Ferretti RE, Leite RE, et al Equal numbers of neuronal and nonneuronal cells make the human brain an isometrically scaled-up primate brain. J Comp Neurol. 2009;513(5):532–41. Epub 2009/02/20. doi: 10.1002/cne.21974 19226510 10.1002/cne.21974

[93] Pakkenberg B, Gundersen HJ. Neocortical neuron number in humans: effect of sex and age. J Comp Neurol. 1997;384(2):312–20. Epub 1997/07/28. 9215725

[94] Pelvig DP, Pakkenberg H, Stark AK, Pakkenberg B. Neocortical glial cell numbers in human brains. Neurobiology of Aging. 2008;29(11):1754–62. doi: 10.1016/j.neurobiolaging.2007.04.013 17544173 10.1016/j.neurobiolaging.2007.04.013

[95] DeFelipe J, Alonso-Nanclares L, Arellano JI. Microstructure of the neocortex: comparative aspects. J Neurocytol. 2002;31(3–5):299–316. 12815249 10.1023/a:1024130211265

[96] Garcia-Cabezas MA, Joyce MP, John Y, Zikopoulos B, Barbas H. Mirror trends of plasticity and stability indicators in primate prefrontal cortex. Eur J Neurosci. 2017;46(8):2392–405. doi: 10.1111/ejn.13706 28921934 10.1111/ejn.13706 PMC5656436

[97] Barbas H. Complementary role of prefrontal cortical regions in cognition, memory and emotion in primates. Adv Neurol. 2000;84:87–110. 11091860

[98] Barbas H. Anatomic basis of cognitive-emotional interactions in the primate prefrontal cortex. Neurosci Biobehav Rev. 1995;19:499–510. 7566750 10.1016/0149-7634(94)00053-4

[99] Penfield W. Neuroglia, normal and pathological In: Penfield W, editor. Cytology and Cellular Pathology of the Nervous System. New York: Paul B. Hoebner; 1932 p. 423–79.

[100] del Río-Hortega P. Rio-Hortega′s Third Contribution to the Morphological Knowledge and Functional Interpretation of the Oligodendroglia Translated by José R. Iglesias-Rozas and Manuel Garrosa. London, UK; Waltham, MA: Elsevier; 1928/2013.

[101] Hellwig B. How the myelin picture of the human cerebral cortex can be computed from cytoarchitectural data. A bridge between von Economo and Vogt. Journal fur Hirnforschung. 1993;34(3):387–402. 8270790

[102] Schuz A, Braitenberg V. The human cortical white matter: quantitative aspects of cortico-cortical long-range connectivity In: Schuz A, Miller R, editors. Cortical areas: unity and diversity. London: Taylor and Francis; 2002 p. 377–85.

[103] Ringo JL, Doty RW, Demeter S, Simard PY. Time is of the essence: a conjecture that hemispheric specialization arises from interhemispheric conduction delay. Cereb Cortex. 1994;4(4):331–43. 7950307 10.1093/cercor/4.4.331

[104] Tomasi S, Caminiti R, Innocenti GM. Areal differences in diameter and length of corticofugal projections. Cereb Cortex. 2012;22(6):1463–72. doi: 10.1093/cercor/bhs011 22302056 10.1093/cercor/bhs011

[105] Innocenti GM, Vercelli A, Caminiti R. The diameter of cortical axons depends both on the area of origin and target. Cereb Cortex. 2014;24(8):2178–88. doi: 10.1093/cercor/bht070 23529006 10.1093/cercor/bht070

[106] Vogt BA. Pain and emotion interactions in subregions of the cingulate gyrus. NatRevNeurosci. 2005;6(7):533–44. 10.1038/nrn1704 PMC2659949 15995724

[107] Barbas H. Pattern in the laminar origin of corticocortical connections. J Comp Neurol. 1986;252:415–22. doi: 10.1002/cne.902520310 3793985 10.1002/cne.902520310

[108] Barbas H, Rempel-Clower N. Cortical structure predicts the pattern of corticocortical connections. Cereb Cortex. 1997;7:635–46. 9373019 10.1093/cercor/7.7.635

[109] Medalla M, Barbas H. Synapses with inhibitory neurons differentiate anterior cingulate from dorsolateral prefrontal pathways associated with cognitive control. Neuron. 2009;61(4):609–20. doi: 10.1016/j.neuron.2009.01.006 19249280 10.1016/j.neuron.2009.01.006 PMC2804928

[110] Barbas H, Medalla M, Alade O, Suski J, Zikopoulos B, Lera P. Relationship of prefrontal connections to inhibitory systems in superior temporal areas in the rhesus monkey. Cereb Cortex. 2005;15(9):1356–70. doi: 10.1093/cercor/bhi018 15635060 10.1093/cercor/bhi018

[111] Bunce JG, Barbas H. Prefrontal pathways target excitatory and inhibitory systems in memory-related medial temporal cortices. NeuroImage. 2011;55(4):1461–74. doi: 10.1016/j.neuroimage.2011.01.064 21281716 10.1016/j.neuroimage.2011.01.064 PMC3500621

[112] Barbas H, Hilgetag CC, Saha S, Dermon CR, Suski JL. Parallel organization of contralateral and ipsilateral prefrontal cortical projections in the rhesus monkey. BMC Neurosci. 2005;6(1):32. 15869709 10.1186/1471-2202-6-32 PMC1134662

[113] Grant S, Hilgetag CC. Graded classes of cortical connections: quantitative analyses of laminar projections to motion areas of cat extrastriate cortex. Eur J Neurosci. 2005;22(3):681–96. doi: 10.1111/j.1460-9568.2005.04232.x 16101750 10.1111/j.1460-9568.2005.04232.x PMC1351360

[114] Hilgetag CC, Grant S. Cytoarchitectural differences are a key determinant of laminar projection origins in the visual cortex. NeuroImage. 2010;51(3):1006–17. Epub 2010 Mar 6. doi: 10.1016/j.neuroimage.2010.03.006 20211270 10.1016/j.neuroimage.2010.03.006

[115] Catani M, Dell′acqua F, Vergani F, Malik F, Hodge H, Roy P, et al Short frontal lobe connections of the human brain. Cortex. 2012;48(2):273–91. Epub 2012/01/03. doi: 10.1016/j.cortex.2011.12.001 22209688 10.1016/j.cortex.2011.12.001

[116] Perge JA, Niven JE, Mugnaini E, Balasubramanian V, Sterling P. Why do axons differ in caliber? J Neurosci. 2012;32(2):626–38. doi: 10.1523/JNEUROSCI.4254-11.2012 22238098 10.1523/JNEUROSCI.4254-11.2012 PMC3571697

[117] Harriger L, van den Heuvel MP, Sporns O. Rich club organization of macaque cerebral cortex and its role in network communication. PLoS ONE. 2012;7(9):e46497 Epub 2012/10/03. doi: 10.1371/journal.pone.0046497 23029538 10.1371/journal.pone.0046497 PMC3460908

[118] Beul SF, Barbas H, Hilgetag CC. A Predictive Structural Model of the Primate Connectome. Scientific reports. 2017;7:43176 doi: 10.1038/srep43176 28256558 10.1038/srep43176 PMC5335700

[119] Thiebaut de Schotten M, Urbanski M, Batrancourt B, Levy R, Dubois B, Cerliani L, et al Rostro-caudal Architecture of the Frontal Lobes in Humans. Cereb Cortex. 2017;27(8):4033–47. doi: 10.1093/cercor/bhw215 27461122 10.1093/cercor/bhw215 PMC6248461

[120] Cerliani L, D′Arceuil H, Thiebaut de Schotten M. Connectivity-based parcellation of the macaque frontal cortex, and its relation with the cytoarchitectonic distribution described in current atlases. Brain Struct Funct. 2017;222(3):1331–49. doi: 10.1007/s00429-016-1280-3 27469273 10.1007/s00429-016-1280-3

[121] Margulies DS, Ghosh SS, Goulas A, Falkiewicz M, Huntenburg JM, Langs G, et al Situating the default-mode network along a principal gradient of macroscale cortical organization. Proc Natl Acad Sci USA. 2016;113(44):12574–9. doi: 10.1073/pnas.1608282113 27791099 10.1073/pnas.1608282113 PMC5098630

[122] Paus T, Castro-Alamancos MA, Petrides M. Cortico-cortical connectivity of the human mid-dorsolateral frontal cortex and its modulation by repetitive transcranial magnetic stimulation. Eur J Neurosci. 2001;14(8):1405–11. 11703468 10.1046/j.0953-816x.2001.01757.x

[123] Koski L, Paus T. Functional connectivity of the anterior cingulate cortex within the human frontal lobe: a brain-mapping meta-analysis. Experimental Brain Research. 2000;133(1):55–65. doi: 10.1007/s002210000400 10933210 10.1007/s002210000400

[124] Cho SS, Strafella AP. rTMS of the Left Dorsolateral Prefrontal Cortex Modulates Dopamine Release in the Ipsilateral Anterior Cingulate Cortex and Orbitofrontal Cortex. PLoS ONE. 2009;4(8):e6725 doi: 10.1371/journal.pone.0006725 19696930 10.1371/journal.pone.0006725 PMC2725302

[125] Ecker C, Andrews D, Dell′Acqua F, Daly E, Murphy C, Catani M, et al Relationship Between Cortical Gyrification, White Matter Connectivity, and Autism Spectrum Disorder. Cereb Cortex. 2016;26(7):3297–309. doi: 10.1093/cercor/bhw098 27130663 10.1093/cercor/bhw098 PMC4898679

[126] McKee AC, Stern RA, Nowinski CJ, Stein TD, Alvarez VE, Daneshvar DH, et al The spectrum of disease in chronic traumatic encephalopathy. Brain. 2013;136(Pt 1):43–64. doi: 10.1093/brain/aws307 23208308 10.1093/brain/aws307 PMC3624697

[127] Braak H, Braak E. Development of Alzheimer-related neurofibrillary changes in the neocortex inversely recapitulates cortical myelogenesis. Acta Neuropathol. 1996;92(2):197–201. 8841666 10.1007/s004010050508

[128] Braak H, Del Tredici K. The preclinical phase of the pathological process underlying sporadic Alzheimer′s disease. Brain. 2015;138(Pt 10):2814–33. doi: 10.1093/brain/awv236 26283673 10.1093/brain/awv236

[129] Mundy P. Annotation: the neural basis of social impairments in autism: the role of the dorsal medial-frontal cortex and anterior cingulate system. J Child PsycholPsychiatry. 2003;44(6):793–809. 10.1111/1469-7610.00165 12959489

[130] Ozonoff S, Pennington BF, Rogers SJ. Executive function deficits in high-functioning autistic individuals: relationship to theory of mind. J Child PsycholPsychiatry. 1991;32(7):1081–105. 10.1111/j.1469-7610.1991.tb00351.x 1787138

[131] GirgiS RR, Minshew NJ, Melhern NM, Nutche JJ, Keshavan MS, Hardan AY. Volumetric alterations of the orbitofrontal cortex in autism. Prog Neuro-Psychoph. 2007;31(1):41–5. 10.1016/j.pnpbp.2006.06.007 PMC2888006 16863674

[132] Courchesne E, Pierce K. Why the frontal cortex in autism might be talking only to itself: local over-connectivity but long-distance disconnection. Current Opinion in Neurobiology. 2005;15(2):225–30. doi: 10.1016/j.conb.2005.03.001 15831407 10.1016/j.conb.2005.03.001

[133] Stoner R, Chow ML, Boyle MP, Sunkin SM, Mouton PR, Roy S, et al Patches of disorganization in the neocortex of children with autism. The New England journal of medicine. 2014;370(13):1209–19. doi: 10.1056/NEJMoa1307491 24670167 10.1056/NEJMoa1307491 PMC4499461

[134] Courchesne E, Mouton PR, Calhoun ME, Semendeferi K, Ahrens-Barbeau C, Hallet MJ, et al Neuron number and size in prefrontal cortex of children with autism. JAMA. 2011;306(18):2001–10. Epub 2011/11/10. doi: 10.1001/jama.2011.1638 22068992 10.1001/jama.2011.1638

[135] Thakkar KN, Polli FE, Joseph RM, Tuch DS, Hadjikhani N, Barton JJ, et al Response monitoring, repetitive behaviour and anterior cingulate abnormalities in autism spectrum disorders (ASD). Brain. 2008;131(Pt 9):2464–78. doi: 10.1093/brain/awn099 18550622 10.1093/brain/awn099 PMC2525446

[136] Kana RK, Keller TA, Cherkassky VL, Minshew NJ, Just MA. Sentence comprehension in autism: thinking in pictures with decreased functional connectivity. Brain. 2006;129(Pt 9):2484–93. Epub 2006/07/13. doi: 10.1093/brain/awl164 16835247 10.1093/brain/awl164 PMC4500127

[137] Kana RK, Keller TA, Minshew NJ, Just MA. Inhibitory control in high-functioning autism: Decreased activation and underconnectivity in inhibition networks. Biological Psychiatry. 2006;62(3):198–206. doi: 10.1016/j.biopsych.2006.08.004 17137558 10.1016/j.biopsych.2006.08.004 PMC4492460

[138] Just MA, Cherkassky VL, Keller TA, Kana RK, Minshew NJ. Functional and anatomical cortical underconnectivity in autism: evidence from an fMRI study of an executive function task and corpus callosum morphometry. Cereb Cortex. 2007;17(4):951–61. doi: 10.1093/cercor/bhl006 16772313 10.1093/cercor/bhl006 PMC4500121

[139] Casanova MF. White matter volume increase and minicolumns in autism. Annals of Neurology. 2004;56(3):453 doi: 10.1002/ana.20196 15349878 10.1002/ana.20196

[140] Anagnostou E, Taylor M. Review of neuroimaging in autism spectrum disorders: what we have learned and where we go from here. Molecular Autism. 2011;2(4):4. 21501488 10.1186/2040-2392-2-4 PMC3102613

[141] Assaf M, Jagannathan K, Calhoun VD, Miller L, Stevens MC, Sahl R, et al Abnormal functional connectivity of default mode sub-networks in autism spectrum disorder patients. Neuroimage. 2010;53(1):247–56. doi: 10.1016/j.neuroimage.2010.05.067 20621638 10.1016/j.neuroimage.2010.05.067 PMC3058935

[142] Medalla M, Barbas H. Specialized prefrontal "auditory fields": organization of primate prefrontal-temporal pathways. Front Neurosci. 2014;8:77 doi: 10.3389/fnins.2014.00077 24795553 10.3389/fnins.2014.00077 PMC3997038

[143] Barbas H, Ghashghaei H, Dombrowski SM, Rempel-Clower NL. Medial prefrontal cortices are unified by common connections with superior temporal cortices and distinguished by input from memory-related areas in the rhesus monkey. J Comp Neurol. 1999;410:343–67. 10404405 10.1002/(sici)1096-9861(19990802)410:3<343::aid-cne1>3.0.co;2-1

[144] Schwartz ML, Goldman-Rakic PS. Callosal and intrahemispheric connectivity of the prefrontal association cortex in rhesus monkey: Relation between intraparietal and principal sulcal cortex. J Comp Neurol. 1984;226:403–20. doi: 10.1002/cne.902260309 6747030 10.1002/cne.902260309

[145] Vogt BA, Pandya DN. Cingulate cortex of the rhesus monkey: II. Cortical afferents. J Comp Neurol. 1987;262:271–89. doi: 10.1002/cne.902620208 3624555 10.1002/cne.902620208

[146] Carmichael ST, Price JL. Connectional networks within the orbital and medial prefrontal cortex of macaque monkeys. J Comp Neurol. 1996;371(2):179–207. 8835726 10.1002/(SICI)1096-9861(19960722)371:2<179::AID-CNE1>3.0.CO;2-#

[147] Wang XJ, Tegner J, Constantinidis C, Goldman-Rakic PS. Division of labor among distinct subtypes of inhibitory neurons in a cortical microcircuit of working memory. Proc Natl Acad Sci USA. 2004;101(5):1368–73. doi: 10.1073/pnas.0305337101 14742867 10.1073/pnas.0305337101 PMC337059

[148] Steele SD, Minshew NJ, Luna B, Sweeney JA. Spatial working memory deficits in autism. JAutism DevDisord. 2007;37(4):605–12. 10.1007/s10803-006-0202-2 16909311

[149] Agam Y, Joseph RM, Barton JJS, Manoach DS. Reduced cognitive control of response inhibition by the anterior cingulate cortex in autism spectrum disorders. Neuroimage. 2010;52(1):336–47. doi: 10.1016/j.neuroimage.2010.04.010 20394829 10.1016/j.neuroimage.2010.04.010 PMC2883672

[150] Koshino H, Kana RK, Keller TA, Cherkassky VL, Minshew NJ, Just MA. fMRI investigation of working memory for faces in autism: visual coding and underconnectivity with frontal areas. Cereb Cortex. 2008;18(2):289–300. doi: 10.1093/cercor/bhm054 17517680 10.1093/cercor/bhm054 PMC4500154

[151] Schipul SE, Keller TA, Just MA. Inter-regional brain communication and its disturbance in autism. Frontiers in Systems Neuroscience. 2011;5:10 doi: 10.3389/fnsys.2011.00010 21390284 10.3389/fnsys.2011.00010 PMC3046360

[152] Buxhoeveden DP, Semendeferi K, Buckwalter J, Schenker N, Switzer R, Courchesne E. Reduced minicolumns in the frontal cortex of patients with autism. Neuropathology and Applied Neurobiology. 2006;32(5):483–91. doi: 10.1111/j.1365-2990.2006.00745.x 16972882 10.1111/j.1365-2990.2006.00745.x

[153] Yip J, Soghomonian JJ, Blatt GJ. Decreased GAD67 mRNA levels in cerebellar Purkinje cells in autism: pathophysiological implications. Acta Neuropathologica. 2007;113(5):559–68. doi: 10.1007/s00401-006-0176-3 17235515 10.1007/s00401-006-0176-3

[154] Mai JK, Majtanik M, Paxinos G. Atlas of the human brain. 4th edition ed. New York: Academic Press—Elsevier; 2015 456 p.

[155] Barbas H, Mesulam MM. Cortical afferent input to the principalis region of the rhesus monkey. Neuroscience. 1985;15:619–37. 4069349 10.1016/0306-4522(85)90064-8

[156] Barbas H. Anatomic organization of basoventral and mediodorsal visual recipient prefrontal regions in the rhesus monkey. J Comp Neurol. 1988;276:313–42. doi: 10.1002/cne.902760302 3192766 10.1002/cne.902760302

[157] Barbas H. Organization of cortical afferent input to orbitofrontal areas in the rhesus monkey. Neuroscience. 1993;56:841–64. 8284038 10.1016/0306-4522(93)90132-y

[158] Barbas H. Pattern in the cortical distribution of prefrontally directed neurons with divergent axons in the rhesus monkey. Cereb Cortex. 1995;5:158–65. 7620292 10.1093/cercor/5.2.158

[159] Germuska M, Saha S, Fiala J, Barbas H. Synaptic distinction of laminar-specific prefrontal-temporal pathways in primates. Cereb Cortex. 2006;16(6):865–75. doi: 10.1093/cercor/bhj030 16151179 10.1093/cercor/bhj030

[160] Medalla M, Lera P, Feinberg M, Barbas H. Specificity in inhibitory systems associated with prefrontal pathways to temporal cortex in primates. Cereb Cortex. 2007;17 Suppl 1:i136–i50. 17725996 10.1093/cercor/bhm068

[161] Medalla M, Barbas H. Anterior cingulate synapses in prefrontal areas 10 and 46 suggest differential influence in cognitive control. J Neurosci. 2010;30(48):16068–81. doi: 10.1523/JNEUROSCI.1773-10.2010 21123554 10.1523/JNEUROSCI.1773-10.2010 PMC3064955

[162] García-Cabezas MA, John YJ, Barbas H, Zikopoulos B. Distinction of Neurons, Glia and Endothelial Cells in the Cerebral Cortex: An Algorithm Based on Cytological Features. Front Neuroanat. 2016;10:107 doi: 10.3389/fnana.2016.00107 27847469 10.3389/fnana.2016.00107 PMC5088408

[163] Zikopoulos B, John YJ, García-Cabezas MA, Bunce JG, Barbas H. The intercalated nuclear complex of the primate amygdala. Neuroscience. 2016;330:267–90. doi: 10.1016/j.neuroscience.2016.05.052 27256508 10.1016/j.neuroscience.2016.05.052 PMC4928580

[164] Gallyas F. Silver staining of myelin by means of physical development. Neurol Res. 1979;1:203–9. 95356 10.1080/01616412.1979.11739553

[165] Peters A, Palay SL, Webster HD. The fine structure of the nervous system Neurons and their supporting cells. 3rd ed New York (NY): Oxford University Press; 1991.

[166] West MJ, Slomianka L, Gundersen HJG. Unbiased stereological estimation of the total number of neurons in the subdivisions of the rat hippocampus using the optical fractionator. Anatomical Record. 1991;231:482–97. doi: 10.1002/ar.1092310411 1793176 10.1002/ar.1092310411

[167] Gundersen HJ. Stereology of arbitrary particles. A review of unbiased number and size estimators and the presentation of some new ones, in memory of William R. Thompson. J Microsc. 1986;143 (Pt 1):3–45. 3761363

[168] Howard CV, Reed MG. Unbiased Stereology, Three-dimensional Measurement in Microscopy. Oxford (UK): BIOS Scientific Publishers Limited; 1998.

[169] Pandya DN, Seltzer B, Barbas H. Input-output organization of the primate cerebral cortex In: Steklis HD, Erwin J, editors. Comparative Primate Biology, Vol 4: Neurosciences. New York (NY): Alan R. Liss; 1988 p. 39–80.

[170] Palomero-Gallagher N, Zilles K. Cortical layers: Cyto-, myelo-, receptor- and synaptic architecture in human cortical areas. Neuroimage. 2017 doi: 10.1016/j.neuroimage.2017.08.035 28811255 10.1016/j.neuroimage.2017.08.035

