# Supplementary material for: Parallel trends in cortical gray and white matter architecture and connections in primates allow fine study of pathways in humans and reveal network disruptions in autism
Source: PLoS Biol. 2018 Feb 5;16(2):e2004559. doi: 10.1371/journal.pbio.2004559 (PMC5814101; doi:10.1371/journal.pbio.2004559)
Supplement: S1 Table — (DOCX) [file pbio.2004559.s002.docx]

**Table S1:** Autism diagnostic interview – revised (ADI-R) scores for autistic subjects (from https://atpportal.org).

| **Human Subject Number** | **AN 06746** | **AN 18892** | **AN 08792** | **AN 07770** | **AN 11989** |
| --- | --- | --- | --- | --- | --- |
| A: Qualitative Impairments in Reciprocal Social Interactions (cutoff: 10) | 26 | 18 | 22 | 12 | 26 |
| B: Qualitative Abnormalities in Communication (Verbal; cutoff: 8) | 18 | 14 | # | 14 | 22 |
| B: Qualitative Abnormalities in Communication (Non-Verbal ; cutoff : 7) | 13 | # | 12 | # | # |
| C: Restricted, Repeated, and Stereotyped Patterns of Behavior (cutoff: 3) | 6 | 6 | 2* | 8 | 12 |
| D: Abnormality of Development Evident at or Before 36 Months (cutoff: 1) | 5 | 3 | 5 | 5 | 5 |
| #: scores were not obtained/not applicable due to lack of communication skills | | | | | |
| *: score was below cutoff threshold due to physical limitations and poor motor skills of donor. However, family members reported repetitive behaviors at a younger age. With this exception, which is not an unusual pattern in the behavioral domain, all donors had difficulties with communication, social behaviors, and atypical interests, consistent with a diagnosis of autism, and the ADI-R scores met and exceeded cutoffs for autism in each of these areas. | | | | | |
