# Supplementary material for: Parallel trends in cortical gray and white matter architecture and connections in primates allow fine study of pathways in humans and reveal network disruptions in autism
Source: PLoS Biol. 2018 Feb 5;16(2):e2004559. doi: 10.1371/journal.pbio.2004559 (PMC5814101; doi:10.1371/journal.pbio.2004559)
Supplement: S2 Table — (DOCX) [file pbio.2004559.s003.docx]

**Table S2:** Data on rhesus macaques, tracers and injection sites used for cortical connectivity analysis and list of quantitative tract-tracing studies that presented this data.

| **Rhesus monkey** | **Injection Site** | **Tracer** | **References with connection data** |
| --- | --- | --- | --- |
|  |  |  |  |
| MDL | ACC 32 | DY | ([1-3](#_ENREF_1)) |
| AK | ACC-32 | DY | ([2-5](#_ENREF_2)) |
| AY | ACC-32 | BDA | ([6-11](#_ENREF_6)) |
| BI | ACC-32 | BDA | ([5](#_ENREF_5), [8-12](#_ENREF_8)) |
| AF | OFC-OPro | HRP-WGA | ([3](#_ENREF_3), [5](#_ENREF_5), [6](#_ENREF_6), [13-16](#_ENREF_13)) |
| AL | OFC-OPro | DY | ([2](#_ENREF_2), [3](#_ENREF_3), [5](#_ENREF_5), [6](#_ENREF_6), [13](#_ENREF_13), [15](#_ENREF_15)) |
| AL | OFC-13 | FB | ([2](#_ENREF_2), [5](#_ENREF_5), [13](#_ENREF_13), [15](#_ENREF_15)) |
| AJ | OFC-13/OPro | FB | ([2](#_ENREF_2), [3](#_ENREF_3), [5](#_ENREF_5), [6](#_ENREF_6), [13](#_ENREF_13), [15](#_ENREF_15)) |
| MBH | LPFC-v46 | HRP-WGA | ([1](#_ENREF_1), [14](#_ENREF_14), [17](#_ENREF_17)) |
| AA | LPFC-v46 | HRP-WGA | ([3](#_ENREF_3), [16](#_ENREF_16), [18](#_ENREF_18)) |
| AD | LPFC-8 | HRP-WGA | ([1](#_ENREF_1), [16](#_ENREF_16), [17](#_ENREF_17)) |
| BF | LPFC-d46 | FB | ([15](#_ENREF_15)) |

**References**

1. Barbas H. Anatomic organization of basoventral and mediodorsal visual recipient prefrontal regions in the rhesus monkey. J Comp Neurol. 1988;276:313-42.

2. Barbas H. Pattern in the cortical distribution of prefrontally directed neurons with divergent axons in the rhesus monkey. Cereb Cortex. 1995;5:158-65.

3. Barbas H, Rempel-Clower N. Cortical structure predicts the pattern of corticocortical connections. Cereb Cortex. 1997;7:635-46.

4. Barbas H, Ghashghaei H, Dombrowski SM, Rempel-Clower NL. Medial prefrontal cortices are unified by common connections with superior temporal cortices and distinguished by input from memory-related areas in the rhesus monkey. J Comp Neurol. 1999;410:343-67.

5. García-Cabezas MA, Barbas H. Anterior Cingulate Pathways May Affect Emotions Through Orbitofrontal Cortex. Cereb Cortex. 2017;27(10):4891-910.

6. Barbas H, Medalla M, Alade O, Suski J, Zikopoulos B, Lera P. Relationship of prefrontal connections to inhibitory systems in superior temporal areas in the rhesus monkey. Cereb Cortex. 2005;15(9):1356-70.

7. Germuska M, Saha S, Fiala J, Barbas H. Synaptic distinction of laminar-specific prefrontal-temporal pathways in primates. Cereb Cortex. 2006;16(6):865-75.

8. Medalla M, Barbas H. Synapses with inhibitory neurons differentiate anterior cingulate from dorsolateral prefrontal pathways associated with cognitive control. Neuron. 2009;61(4):609-20.

9. Medalla M, Barbas H. Anterior cingulate synapses in prefrontal areas 10 and 46 suggest differential influence in cognitive control. J Neurosci. 2010;30(48):16068-81.

10. Bunce JG, Barbas H. Prefrontal pathways target excitatory and inhibitory systems in memory-related medial temporal cortices. NeuroImage. 2011;55(4):1461-74.

11. Bunce JG, Zikopoulos B, Feinberg M, Barbas H. Parallel prefrontal pathways reach distinct excitatory and inhibitory systems in memory-related rhinal cortices. J Comp Neurol. 2013;512(18):4260-83.

12. Medalla M, Lera P, Feinberg M, Barbas H. Specificity in inhibitory systems associated with prefrontal pathways to temporal cortex in primates. Cereb Cortex. 2007;17 Suppl 1:i136-i50.

13. Barbas H. Organization of cortical afferent input to orbitofrontal areas in the rhesus monkey. Neuroscience. 1993;56:841-64.

14. Rempel-Clower NL, Barbas H. The laminar pattern of connections between prefrontal and anterior temporal cortices in the rhesus monkey is related to cortical structure and function. Cereb Cortex. 2000;10(9):851-65.

15. Barbas H, Hilgetag CC, Saha S, Dermon CR, Suski JL. Parallel organization of contralateral and ipsilateral prefrontal cortical projections in the rhesus monkey. BMC Neurosci. 2005;6(1):32.

16. Hilgetag CC, Medalla M, Beul S, Barbas H. The primate connectome in context: principles of connections of the cortical visual system. NeuroImage. 2016;134:685-702.

17. Medalla M, Barbas H. Diversity of laminar connections linking periarcuate and lateral intraparietal areas depends on cortical structure. Eur J Neurosci. 2006;23(1):161-79.

18. Barbas H, Mesulam MM. Cortical afferent input to the principalis region of the rhesus monkey. Neuroscience. 1985;15:619-37.
