# Supplementary material for: Parallel trends in cortical gray and white matter architecture and connections in primates allow fine study of pathways in humans and reveal network disruptions in autism
Source: PLoS Biol. 2018 Feb 5;16(2):e2004559. doi: 10.1371/journal.pbio.2004559 (PMC5814101; doi:10.1371/journal.pbio.2004559)
Supplement: S3 Table — (DOCX) [file pbio.2004559.s004.docx]

**Table S3:** Cortical areas grouped by lobe and structural type that are connected with ACC, OFC and LPFC, based on tract-tracing studies in rhesus macaques.

| **Lobe / Structural Type** | **Cortical Area** | **Presence of connection with (+)** | | |
| --- | --- | --- | --- | --- |
|  |  | **ACC**  **(32)** | **pOFC**  **(OPro, 13)** | **LPFC**  **(46, 8)** |
| **Frontal** |  |  |  |  |
| ***Agranular*** | MPAll | + | + |  |
|  | OPAll | + | + |  |
|  | 24a | + | + |  |
| ***Dysgranular*** | 24b | + | + |  |
|  | 24c | + | + |  |
|  | 25 | + | + |  |
|  | 32 | + | + |  |
|  | OPro | + | + |  |
|  | 13 | + | + |  |
|  | Pro M |  | + |  |
| ***Eulaminate*** | 9 | + | + | + |
|  | 10 | + | + | + |
|  | 11 | + | + |  |
|  | 14 | + | + |  |
|  | 12 | + | + | + |
|  | 46 | + | + | + |
|  | 8 |  |  | + |
|  | 6DC |  |  | + |
|  | 6DR |  |  | + |
| **Temporal** *(including Insula)* |  |  |  |  |
| ***Agranular*** | TPAll | + | + |  |
|  | APAll | + | + |  |
|  | Iag | + | + |  |
|  | 28 | + |  |  |
| ***Dysgranular*** | TPro | + | + |  |
|  | APro |  |  |  |
|  | 35/36 (rostral) | + | + |  |
|  | TH/TF (rostral) | + | + |  |
|  | paAr | + |  |  |
|  | Idg | + | + |  |
| ***Eulaminate*** | TE1, 2, 3 | + | + | + |
|  | TEa | + |  | + |
|  | TEm |  |  | + |
|  | IPa | + |  |  |
|  | PGa | + |  | + |
|  | TPO | + | + | + |
|  | TS1, 2, 3 | + | + | + |
| **Parietal / Occipital** |  |  |  |  |
| ***Dysgranular*** | ProStr | + |  |  |
|  | 23 | + | + | + |
| ***Eulaminate*** | PO | + |  | + |
|  | PG |  |  | + |
|  | IPd |  |  | + |
|  | Central Op |  |  | + |
|  | V2 |  |  | + |
|  | V3 |  |  | + |
|  | V4 |  |  | + |
|  | MST |  |  | + |
|  | MT |  |  | + |
